# Transport signatures of gate-tunable topological phase transition in ultrathin *β*-Ag_2_Te

**DOI:** 10.1093/nsr/nwag229

**Published:** 2026-04-17

**Authors:** Wei Ai, Xiao-Feng Luo, Zhaochao Liu, Ying Deng, Zunxian Lv, Yuyu He, Lingyue Li, Xuewen Fu, Feng Luo, Jingyue Wang, Jin-Zhu Zhao, Jinxiong Wu

**Affiliations:** Tianjin Key Lab for Rare Earth Materials and Applications, Center for Rare Earth and Inorganic Functional Materials, Collaborative Innovation Center of Materials Science, School of Materials Science and Engineering, Academy for Advanced Interdisciplinary Studies, Nankai University, Tianjin 300350, China; Guangdong Basic Research Center of Excellence for Structure and Fundamental Interactions of Matter, Guangdong Provincial Key Laboratory of Quantum Engineering and Quantum Materials, School of Physics, South China Normal University, Guangzhou 510006, China; Guangdong-Hong Kong Joint Laboratory of Quantum Matter, Frontier Research Institute for Physics, South China Normal University, Guangzhou 510006, China; Tianjin Key Lab for Rare Earth Materials and Applications, Center for Rare Earth and Inorganic Functional Materials, Collaborative Innovation Center of Materials Science, School of Materials Science and Engineering, Academy for Advanced Interdisciplinary Studies, Nankai University, Tianjin 300350, China; Ultrafast Electron Microscopy Laboratory, Key Laboratory of Weak-Light Nonlinear Photonics, School of Physics, Nankai University, Tianjin 300071, China; Tianjin Key Lab for Rare Earth Materials and Applications, Center for Rare Earth and Inorganic Functional Materials, Collaborative Innovation Center of Materials Science, School of Materials Science and Engineering, Academy for Advanced Interdisciplinary Studies, Nankai University, Tianjin 300350, China; Tianjin Key Lab for Rare Earth Materials and Applications, Center for Rare Earth and Inorganic Functional Materials, Collaborative Innovation Center of Materials Science, School of Materials Science and Engineering, Academy for Advanced Interdisciplinary Studies, Nankai University, Tianjin 300350, China; Tianjin Key Lab for Rare Earth Materials and Applications, Center for Rare Earth and Inorganic Functional Materials, Collaborative Innovation Center of Materials Science, School of Materials Science and Engineering, Academy for Advanced Interdisciplinary Studies, Nankai University, Tianjin 300350, China; Ultrafast Electron Microscopy Laboratory, Key Laboratory of Weak-Light Nonlinear Photonics, School of Physics, Nankai University, Tianjin 300071, China; Tianjin Key Lab for Rare Earth Materials and Applications, Center for Rare Earth and Inorganic Functional Materials, Collaborative Innovation Center of Materials Science, School of Materials Science and Engineering, Academy for Advanced Interdisciplinary Studies, Nankai University, Tianjin 300350, China; Shandong Key Laboratory of Intelligent Energy Materials, School of Materials Science and Engineering, China University of Petroleum (East China), Qingdao 266580, China; Guangdong Basic Research Center of Excellence for Structure and Fundamental Interactions of Matter, Guangdong Provincial Key Laboratory of Quantum Engineering and Quantum Materials, School of Physics, South China Normal University, Guangzhou 510006, China; Guangdong-Hong Kong Joint Laboratory of Quantum Matter, Frontier Research Institute for Physics, South China Normal University, Guangzhou 510006, China; Center for Computational Science and Engineering, Southern University of Science and Technology, Shenzhen 518055, China; Tianjin Key Lab for Rare Earth Materials and Applications, Center for Rare Earth and Inorganic Functional Materials, Collaborative Innovation Center of Materials Science, School of Materials Science and Engineering, Academy for Advanced Interdisciplinary Studies, Nankai University, Tianjin 300350, China

**Keywords:** topological phase transition, gate-tunable, Stark effect, topological insulator, Berry phase

## Abstract

The topological phase transition (TPT) between trivial and nontrivial quantum states has attracted significant research interests due to its fundamental importance in condensed matter physics and potential applications in next-generation low-power electronics. However, previous TPT has mainly been achieved by structural modification and chemical doping on topological materials, which presents substantial challenges for practical device applications. Here, we report the direct observation of device-level reversible TPT in *β*-Ag_2_Te nanoflakes controlled by an out-of-plane gate voltage. The quantum oscillations in *β*-Ag_2_Te exhibit a π Berry phase when applying a small top-gate voltage, while displaying a topologically trivial Berry phase under a high gate voltage. Theoretical calculations reveal that this electrically induced TPT originates from the Stark-effect-mediated band structure modification under external electric fields. Based on this gate-tunable TPT in *β*-Ag_2_Te, we successfully fabricate the preliminary prototype device of TPT field-effect transistor, exhibiting a high current on–off ratio exceeding 10^4^. This work establishes an electric-field control paradigm for topological phase engineering, and builds a crucial bridge between fundamental topological physics and novel device concepts.

## Teaser

Gate-voltage-driven reversible topological phase transition with π Berry phase switching is achieved in β-Ag2Te nanoflakes, enabling a prototype topological phase-transition field-effect transistor with an on–off ratio exceeding 104.

## INTRODUCTION

Phase transitions are central to condensed matter physics, with controllable transitions being of particular scientific and technological importance. Unlike conventional structural phase transitions, the topological phase transition (TPT) involves a change in the band structure’s topology while leaving the crystal structure intact, offering superior reversibility [1–3]. Hence, the phase transition between non-trivial and trivial topological states holds great significance for both basic materials science and next-generation low-power electronic devices [4–6]. Over the past decade, significant efforts have been devoted to realizing TPT through diverse approaches such as elemental doping [7–10], magnetic field [11–14], ultrafast photoexcitation [15,16], high pressure [17–19] and scanning tunneling microscope tip-induced modulation [20]. While these methods can effectively manipulate topological states, their practical adoption in functional devices remains hindered by intrinsic limitations, including irreversibility, structural invasiveness, and incompatibility with conventional semiconductor architectures. For TPT’s transition from laboratory curiosities to practical applications, a critical step lies in achieving device-level electrical control of topological states via gate voltage—a cornerstone of modern electronics.

In principle, applying external electric fields on low-dimensional quantum materials can induce the quantum-confined Stark effect (QCSE) [21–24], subtly reshaping the band structure of topological materials and potentially driving reversible TPT. Compared to other modulation strategies of TPT, gate control combines non-destructive operation, high tunability, and seamless integration with existing semiconductor

processes. However, this approach imposes stringent material requirements: the candidate system should exhibit gate-tunable topological states sensitive to moderate electric fields, robust topological properties resilient against interfacial disorders, and the compatibility with conventional device fabrication. Despite theoretical predictions of gate-voltage-induced TPT and TPT-based transistors were proposed [4,5,23,25], experimental validation at the device level—particularly with direct transport signatures—remains elusive, impeding progress toward topological phase transition field-effect transistors (TPT-FET).

Here, we demonstrate gate-voltage-driven reversible TPT in *β*-Ag_2_Te nanoflakes through combined quantum transport measurements and *ab initio* calculations. Based on the TPT in *β*-Ag_2_Te grown by chemical vapor deposition (CVD), a topological FET were successfully fabricated with an on/off ratio of about 10^4^, which is the first gate-tunable TPT-FET device. Our work successfully integrates the manipulation of a TPT into a standard top-gate field-effect device architecture. This represents a significant conceptual advance, providing a direct platform to control and probe topological states at the device level.

## RESULTS AND DISCUSSION

### Band structure analysis and sample preparation

*β*-Ag_2_Te, a classic thermoelectric material [26], first gained significant attention for the observation of a large linear magnetoresistance in its polycrystalline films [27,28]. This intriguing phenomenon prompted deeper investigations, which ultimately established monoclinic *β*-Ag_2_Te as a narrow-gap three-dimensional topological insulator by both theoretical calculations and experimental studies [29–31]. Recently, this material continues to be of great interest due to its exceptional transport and optical properties, including high carrier mobility and strong anisotropy [32–36].

**Figure 1.**
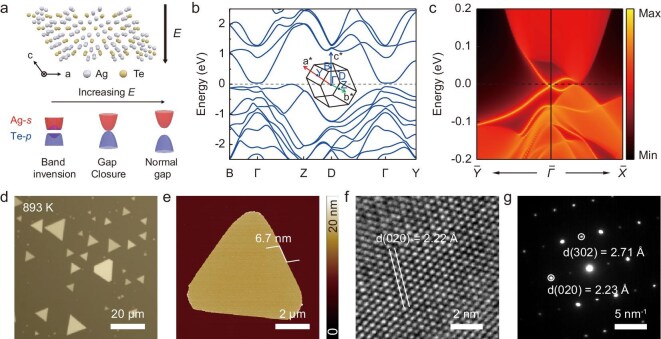
Band structure analysis and sample preparation. (a) Illustration of Stark-effect-induced TPT in the ultrathin *β*-Ag_2_Te crystals with the external electric field along (001) crystallographic plane. (b) The calculated electronic band structure of *β*-Ag_2_Te, showing a tiny band gap of 36.7 meV. Inset: The Brillouin zone with labeled high-symmetry points. (c) Surface state cutting along (001) crystallographic plane, confirming the existence of topologically protected conducting states near the Fermi level. (d) OM image of as-grown *β*-Ag_2_Te nanosheets on mica, which were synthesized at a relatively low temperature (893 K). (e) AFM image of a 6.7-nm-thick *β*-Ag_2_Te nanoflake, showing an ultra-smooth surface. (f and g) High-resolution TEM image (f) and corresponding selected-area electron diffraction pattern (g) of the *β*-Ag_2_Te nanoflake.

The QCSE describes the change in the energy levels of low-dimensional quantum systems under an applied external electric field. In such low-dimensional structures, the electric field induces a Stark shift of the electronic bands. As shown in Fig. 1a, the topological phase of *β*-Ag_2_Te is determined by the band inversion between orbitals of different parities (specifically, Ag-*s* and Te-*p* orbitals). In the nontrivial topological state, the strong spin–orbit coupling (SOC) effect in *β*-Ag_2_Te makes the energy level of the originally lower Te *p*-orbital above that of the Ag *s*-orbital, resulting in a band inversion and topological surface state. A strong vertical electric field applied perpendicular to the nano-flake plane induces an orbital-dependent Stark shift, causing the energy levels to shift in opposite directions due to their distinct spatial distributions and orbital characters. When the electric field strength reaches a critical value, this relative energy shift is sufficient to drive the originally inverted band structure to a structure with a normal band gap (Ag-*s* orbital above the Te-*p* orital). This changes the topological invariant (for example, the *Z*_₂_ index), driving the system from a topological insulator to a trivial insulator (or vice versa). At the critical point of this transition, the band gap closes.

For the QCSE to effectively control a TPT, several conditions must be met: (i) given the limited strength of gate fields, the topological material should be naturally close to a phase transition point, making it highly susceptible to external tuning. (ii) The material must be sufficiently thin to allow a substantial potential drop across it with an achievable gate voltage, enabling significant modification of the band structure for various intriguing phenomena. (iii) The material must be stable and of high quality to maintain high carrier mobility even after device fabrication. This is essential for observing quantum oscillations (SdH oscillations) and precisely detecting the change in Berry phase, which serves as the signature of the TPT at the device level.

**Figure 2.**
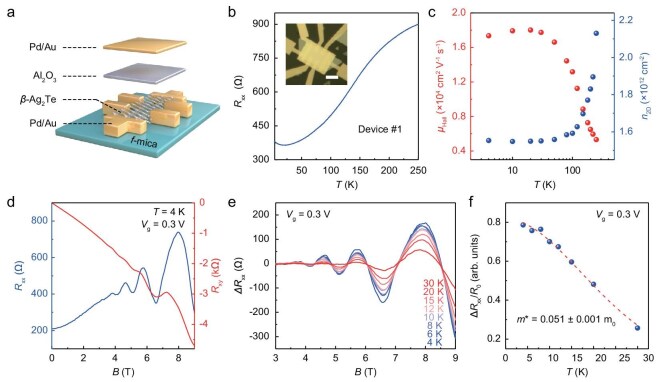
Electrical transport and Shubnikov-de Haas quantum oscillations in the as-synthesized ultrathin *β*-Ag_2_Te crystal of device #1. (a) Schematic diagram of the *β*-Ag_2_Te top-gate Hall device, which were directly fabricated on mica substrate. The Pd/Au (5 nm/30 nm) serves as the contact electrodes and the top-gate electrode, and 12-nm-thick Al_2_O_3_ film grown by ALD was used as the top-gate dielectric. (b) Longitudinal resistance (*R*_xx_) as a function of temperature from 4 to 250 K, showing a metallic behavior upon cooling down. Inset, OM image of six-terminal *β*-Ag_2_Te Hall device with a thickness of 10.1 nm. Bar: 10 μm. (c) The extracted Hall mobility and carrier density as a function of temperature. (d) Longitudinal resistance (*R*_xx_) and transverse resistance (*R*_xy_) as a function of the applied perpendicular magnetic field measured at 4 K. (e) Temperature-dependent SdH oscillations amplitude (subtracting a smooth background) from 4 to 30 K. (f) Temperature-dependent Δ*R*_xx_/*R*_0_ values of the SdH oscillations and Lifshitz–Kosevish formula fitting at *B* = 7.9 T, showing a very small effective mass of 0.051 ± 0.001 *m*_0_.

Fortunately, *β*-Ag_2_Te satisfies all three aforementioned conditions. First, as confirmed by the recalculated bulk band structure (Fig. 1b), *β*-Ag_2_Te is indeed a three-dimensional topological insulator with a narrow bulk band gap of ∼36.7 meV, whose nontrivial topological nature (*Z*_2_ = 1) is confirmed by analyzing the parities of occupied wave functions for time-reversal-invariant points in the Brillouin zone. The topological surface states of *β*-Ag_2_Te on the (001) surface were investigated by projecting the wavefunctions on Wannier basis via tight-binding approximations [37], which revealed topologically protected conducting states near the Fermi level (Fig. 1c). Second, we can achieve the controllable growth of *β*-Ag_2_Te nanoflakes with thicknesses <10 nm to avoid the screening effect of electric field in bulk samples. On the basis of our previous research on the Bi_2_O_3_-assisted synthesis of *β*-Ag_2_Te nanoplates [36], the thinner *β*-Ag_2_Te nanoplates (domain size ∼20 μm) exhibited triangular-like or hexagonal-like morphologies on the mica substrate by lowering the deposition temperature. As shown in Fig. 1d, the typical thickness of *β*-Ag_2_Te nanoflakes grown at low temperature (893 K) is <20 nm (Fig. S1), which is apparently thinner than that deposited at high temperature (953 K) with a thickness varying from 60 to 180 nm (Fig. S2). The thinnest *β*-Ag_2_Te nanoplate was around 6.7 nm with an atomically flat surface (Fig. 1e). The capability of growing ultrathin *β*-Ag_2_Te sample (<15 nm) provides the key foundation for the successful observation of gate-induced TPT, which is distinct from previous reports regarding transport behaviors on ∼100-nm-thick *β*-Ag_2_Te sample with limited gate tunability [32,38]. The crystalline phase of CVD-grown *β*-Ag_2_Te was confirmed by transmission electron microscope (TEM) imaging along (001) atomic plane (Fig. 1f and g). Finally, the as-grown thin flakes exhibit ultrahigh carrier mobility, which enables the observation and analysis of the Berry phase of quantum oscillations therein (For details, see Fig. 2). We emphasize that due to the non-trivial band topology, an electron wave function acquires an additional Berry phase of π upon completing a closed orbit. This Berry phase directly manifests in the phase of quantum oscillations, making phase analysis a straightforward method for its determination. It has been successfully shown in topological materials [39–41] and extensively used ever since.

### High mobility and SdH oscillations in ultrathin *β*-Ag_2_Te nanoplates

To evaluate the basic transport behaviors of CVD-grown ultrathin *β*-Ag_2_Te crystals, we directly fabricated standard top-gate Hall bar devices on mica without specific protection from H_2_O/O_2_. Figure 2a illustrates the device configuration for electrical measurements, in which the Pd/Au (5 nm/30 nm) serves as the contact electrodes and the top-gate electrode, and 12-nm-thick Al_2_O_3_ film grown by atomic layer deposition (ALD) was used as the top-gate dielectric. As shown in Fig. 2b, a metallic longitudinal resistance versus temperature (*R*_xx_-*T*) curve can be observed in a 10.1-nm-thick *β*-Ag_2_Te nanoflake (named device #1, Fig. S3), which can be attributed to a rapid decrease in carrier concentration accompanied by a sharp increase in mobility during the cooling process (Fig. 2c). Notably, when no gate voltage was applied, the Hall mobility of the CVD-grown *β*-Ag_2_Te nanoflakes can be as high as 17 354 cm^2^ V^−1^ s^−1^ with a low carrier density of about 1.55 × 10^12^ cm^−2^ at 4 K (Fig. 2c). The high mobility nature makes *β*-Ag_2_Te an ideal platform to study quantum oscillations and the accompanied Berry phase. On the other hand, the low carrier density guarantees the efficient electrostatic control over the channel even by a small external gate voltage, which is totally different from the well-known Bi_2_Se_3_ topological insulator with high residual carrier density [42]. As shown in Fig. 2d, pronounced SdH oscillations and corresponding plateau-like signatures in Hall resistance (*R*_xy_) were observed under an increasing magnetic field of less than 9 T, suggesting the high quality of the CVD samples. Based on the analysis of temperature-dependent SdH oscillations amplitude ∆*R*_xx_ (Fig. 2e), the carrier effective mass m* of as-grown *β*-Ag_2_Te nanoflakes could be extracted. The m* was estimated by fitting the temperature-dependent oscillation amplitudes with the Lifshitz–Kosevich formula [43]


(1) \begin{eqnarray*}
\Delta {R}_{xx} = 4{R}_0{e}^{\frac{{ - 4{\pi }^3{k}_B{T}_D}}{{h{\omega }_c}}}\frac{{\frac{{4{\pi }^3{k}_B{T}_D}}{{h{\omega }_C}}}}{{{\mathrm{sinh}} \left( {\frac{{4{\pi }^3{k}_B{T}_D}}{{h{\omega }_C}}} \right)}},
\end{eqnarray*}

where *h* is Planck’s constant, *T*_D_ is the dingle temperature, *k*_B_ is the Boltzmann constant, *R*_0_ is the longitudinal resistance at 0 T, and ${{{\omega }}}_{{C}} = \frac{{{{eB}}}}{{{{{m}}}^{{*}}}}$ is the cyclotron frequency. The temperature-dependent SdH amplitudes at 7.9 T gave an m* as small as 0.051 ± 0.001 m_0_. Such a small electron mass indicates a sharp energy dispersion of conduction band in *β*-Ag_2_Te, and agrees well with previous theoretical and experimental reports [30,32–34]. Moreover, we performed the FFT analysis of the quantum oscillations in Fig. 2e, showing a one-band oscillatory behavior with fundamental (1st) frequency and related 2nd and 4th harmonics (Fig. S4).

### Gate-tunable TPT in ultrathin *β*-Ag_2_Te nanoplates

**Figure 3.**
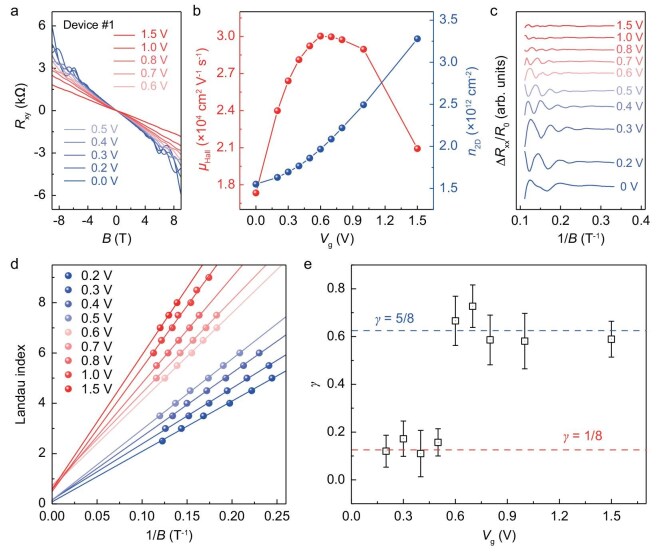
Gate-tunable TPT in ultrathin *β*-Ag_2_Te crystal. (a) Gate-dependent Hall measurements at 4 K of device #1. The apparent decrease of linear slope indicates the increased carrier density and efficient electrical control by gating. (b) The extracted Hall mobility and carrier density as a function of gate voltage, showing a maximum of ∼30 000 cm^2^ V^−1^ s^−1^ at *V*_g_ = 0.6 V. (c) A stacking view of SdH oscillations amplitude as a function of 1/B at various gate voltage. (d) Landau fan diagram at various top-gate voltage. Where an integer Landau index is assigned to the peak of SdH oscillation and a half-integer is assigned to the valley. (e) The intercept *γ* of Landau fan diagram as a function of top-gate voltage. A sudden change from 1/8 to 5/8 occurs at a critical voltage, indicating a clear topological phase transition.

The gate tunability of quantum oscillations serves as a powerful tool for exploring emergent quantum phenomena, such as TPT. To systematically investigate this tunability, we further performed detailed Hall measurements (Fig. 3a) at various top-gate voltages. As shown in Fig. 3a, the slopes of the Hall curves can be effectively tuned by the top gate, indicating the high tunability of the charge carriers in ultrathin *β*-Ag_2_Te nanoplates. When the gate voltage increased from 0 to 1.5 V, the carrier density, *n*_2D_ = 1/*ke*_0_ (where *k* is slope of the Hall curve and *e*_0_ is the elemental charge), varied from 1.5 × 10^12^ to 3.3 × 10^12^ cm^−2^ (Fig. 3b). Meanwhile, the device maintains high carrier mobility and reaches the highest value of about 30 000 cm^2^/Vs. Here, the Hall mobility was extracted using the low-field linear approximation, which minimizes the influence of high-field non-linearity on the extracted mobility values. The observed reduction in mobility at higher gate voltages in Fig. 3b can be attributed to a transition from dominated ionized impurity scattering due to insufficient screening at low concentrations, to dominated interface scattering and high-density effects at high concentrations [44]. Importantly, the quantum oscillations in *R*_xx_ can also be effectively regulated when changing the top-gate voltages (Fig. 3c and Fig. S5). Besides, the increasing period of SdH oscillations indicates an increase of Fermi energy when increasing the top-gate voltage, thus the Fermi energy can be effectively controlled in the devices.

**Figure 4.**
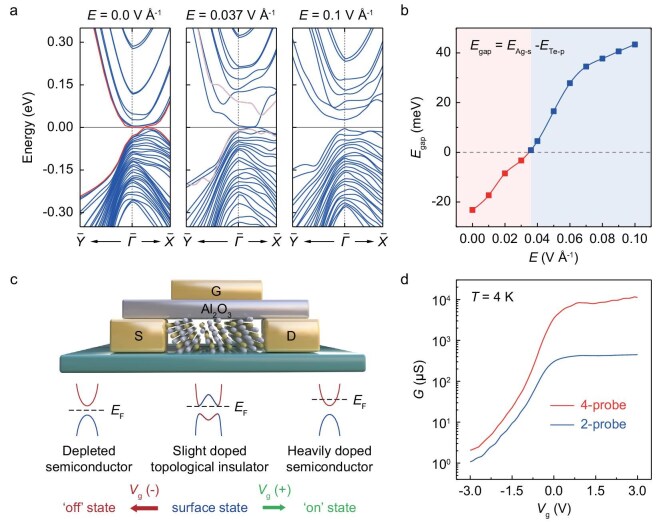
Theoretical origin for the observed gate-tunable TPT and Ag_2_Te-based TPT transistor of device #1. (a) The band structure and surface state for a 12-unicell thickness slab of *β*-Ag_2_Te. These insets from left to right are the band for 0.0, 0.037, and 0.1 eV/Å electric field applied along the [001] direction. The red color refers to surface states that are topologically protected and the light pink color refers to the one without topological properties after energy band inversion. (b) Band gap change under electric field. The band gap values are defined as the energy difference between the band occupied by Ag-*s* and Te-*p* orbitals. The red and blue background refer to topologically nontrivial and trivial state, respectively. (c) Diagram of TPT transistor with ultrathin *β*-Ag_2_Te as channel material in various state. At zero gate voltage, the system behaves as a lightly doped topological insulator. Upon applying a positive gate voltage, it transitions into a heavily doped semiconductor, corresponding to the on-state of the device. Under a negative gate voltage, the system enters a turned-off semiconductor state, representing the off-state of the device. (d) The comparison of 2-probe and 4-probe transfer curves of the *β*-Ag_2_Te-based TPT transistor at 4 K. The 4-probe transfer curve reveals a sharp current switching behavior with a high 4-probe field-effect mobility of ∼22 100 cm^2^ V^−1^ s^−1^ and an on–off ratio of ∼10⁴.

For Dirac fermions, an additional nontrivial Berry phase of π was produced to the electron wave function when it completes a closed orbit because of the chirality. The additional Berry phase of π naturally leads a uniform phase shift to the SdH oscillations, which has been proved in the graphene [45,46], topological materials [47], semiconductor [48,49], and so on. Therefore, the phase analysis of quantum oscillations is a reliable approach to the determination of Berry phase. To extract the Berry phase of the CVD-grown ultrathin *β*-Ag_2_Te nanoflakes at various gate voltages, a Landau fan diagram (Fig. 3d) was plotted based on the Lifshitz–Onsager quantization rule: $\frac{{{{{A}}}_{{F}}}}{{{B}}} = {{n}} + \frac{1}{2} - \frac{{{{{\Phi }}}_{{B}}}}{{2{{\pi }}}} - {{\delta }} = {{n}} + {{\gamma }}$, where *n* is the Landau level index, *γ* is the intercept, ${{{\Phi }}}_{{B}}$ represents Berry phase. In the Landau plots, the integer indices represent the resistance peaks in 1/*B*, while the half-integer indices are assigned to the valleys. In 3D systems, Fermi surface curvature introduces an additional phase shift *δ* = 1/8. Consequently, intercepts of 1/8 and 5/8 at 1/*B* = 0 characterize 3D topological nontrivial and trivial bands, respectively. Figure 3e displays the top-gate-dependent intercepts *γ* derived from linear extrapolation of Landau indices to 1/*B* = 0. Notably, a sharp transition in *γ* from 1/8 to 5/8 emerges at critical gating. The intercept *γ* remains near 1/8 when *V*_g_ < 0.6 V, consistent with a 3D topological nontrivial state. However, the intercept abruptly shifts to around 5/8 when the top-gate voltage was applied above 0.6 V, suggesting a trivial quantum phase therein. The discontinuous evolution of Berry phase undoubtedly demonstrates a gate-induced TPT in high-mobility *β*-Ag_2_Te thin flakes, which was further confirmed by the peak-to-valley transition analysis upon gating (Fig. S6). Similar results were observed on two other *β*-Ag_2_Te top-gate devices with thicknesses of 11.1 nm (named device #2, Figs S7–S10) and 13.9 nm (named device #3, Figs S11 and S12). Notably, a sharp transition in *γ* from 1/8 to 5/8 emerges at critical gating, regardless of the detailed assignment of Landau levels or the specific fitting window (see Fig. S13). Moreover, as evidenced by FFT analysis (Fig. S14), single-band transport is observed both before and after the phase transition. Combining with discontinuous change of Berry phase and high switching ratio of up to 10⁴ (Fig. 4), the modification in Berry phase can be ascribed to the band inversion rather than multi-band effects, or Fermi level crossing different regions of the band structure. Furthermore, the angular-dependent Hall measurements show a 2D character of quantum oscillations (Fig. S15). Moreover, the thicker sample shows a larger critical gate voltage, which is primarily due to the screening effect of the electric field within the sample (Fig. S16).

### Theoretical origin and TPT-based transistor

To reveal the origin of the TPT in *β*-Ag_2_Te, *ab initio* calculations were employed. We firstly performed band structure calculations in a 12-unit-cell-thick *β*-Ag_2_Te slab (∼10 nm), whose thickness is almost consistent with our experimental devices. As shown in the left panel of Fig. 4a, the slab shows an inverted band gap of about 23.2 meV with a topologically protected surface state (see the red curves in Fig. 4a) when no external electric field was applied. Importantly, the band gap almost vanishes to zero (∼1.2 meV) when applying an electric field of *E* = 0.037 eV/Å along (001) direction (Fig. 4b). This indicates the system is approaching to the critical TPT point between a topological insulator and a semiconductor. With increasing the electric field to *E* = 0.1 eV/Å on the slab, a sizable gap of ∼43.3 meV can be re-opened in *β*-Ag_2_Te slab. The disappearance of in-gap surface states can verify its trivial semiconducting nature of the band structure. Figure 4b plotted the extracted energy gaps of the slab as a function of out-of-plane electric fields. This dynamic closure and reopening of bandgap are linked to the disappearance of in-gap surface states (red curves in Fig. 4a), suggesting an electric-field-induced transition from a nontrivial *Z*_₂_ = 1 phase to a trivial *Z*_₂_ = 0 phase [20,50]. It’s worth noting that the observed bandgap changes of several tens meV at *E* = 0.1 eV/Å are reasonable and consistent with the previously reported Stark effect in narrow-band-gap quantum materials [22,51]. Here, the band gap is defined as the energy difference between the band occupied by Ag-*s* and Te-*p* orbitals. To this end, the negative band gap suggests the non-trivial band structure in *β*-Ag_2_Te with band inversion between conduction and valence bands, whereas the positive band gap corresponds to the trivial semiconducting band structure. Thus, the theoretical calculations align well with the experimental results, in which TPT can be achieved by applying vertical gate voltages in *β*-Ag_2_Te thin films (Fig. 3). It should be noted that the *E*-field included density functional theory (DFT) method used in our work for band structure calculation is inherently incompatible with periodic potential boundary conditions, making it only suitable for qualitative analysis on the evolution of band structures with respect to external *E*-field.

To demonstrate the potential of *β*-Ag_2_Te for future dissipation-free electronics, we simultaneously measure 2-probe and 4-probe transfer characteristics in top-gated devices using ultrathin *β*-Ag_2_Te as the channel material. Here, we should emphasize that the top-gate FET device in Fig. 4 is the same one with Fig. 2 and Fig. 3. Remarkably, the 4-probe transfer curve (Fig. 4d, Figs S10 and S12) reveals a sharp current switching behavior with an on–off ratio of ∼10⁴—a performance unprecedented in conventional topological insulators with in-gap metallic surface states. A slight clockwise hysteresis is observed during our gate voltage sweep, which can be attributed to the presence of interfacial charge traps between the ALD-grown Al_2_O_3_ and *β*-Ag_2_Te interface (Fig. S17). This exceptional gate controllability can be ascribed to the gate-voltage-driven TPT in *β*-Ag_2_Te-based topological transistor. As illustrated in Fig. 4c, the non-gated nanosheet is initially a slightly doped topological insulator with the Fermi level located near the bottom of the conduction band. As a positive gate voltage is applied, the carrier concentration increases, pushing the Fermi level further into the conduction band. Once the gate voltage exceeds the critical value for the TPT, the system changes from a topological insulator to a heavily doped semiconductor, leading to an ‘On-state’. Conversely, when a negative gate voltage is applied, the Fermi level shifts downward toward the intrinsic band gap. Under a sufficiently strong negative gate voltage that induces the TPT, the Fermi level moves into the induced band gap. This results in a significant reduction in carrier concentration, thereby revealing an insulating state (‘Off-state’). Furthermore, by linear fitting of the transfer curve based on the equation of ${{{\mu }}}_{{{app}}} = \frac{{{L}}}{{{{W}}{{{C}}}_{{g}}}}\frac{{{{d}}{{{I}}}_{{{ds}}}}}{{{{d}}{{{V}}}_{{g}}}}$, the 4-probe field-effect mobility can be estimated as high as 22 100 cm^2^ V^−1^ s^−1^, which matches well with the mobility derived from the Hall measurements Fig. 3b. Our *β*-Ag_2_Te-based TPT-FET shows a distinct underlying physical mechanism with the one of Fermi level shifting tuned by ionic liquid gate [52]. However, we should admit that our *β*-Ag_2_Te-based TPT-FET is a preliminary prototype device, which still leaves considerable room for improvement in terms of switching ratio and other electrical performance metrics.

## CONCLUSIONS

In summary, we achieved the direct observation of gate-tunable TPT on *β*-Ag_2_Te top-gate Hall bar device by quantum transport measurements and Berry phase analysis. Theoretical calculations reveal that such TPT in *β*-Ag_2_Te crystals originates from the Stark-effect-induced regulation of band structures. A small external electric field along (001) crystallographic plane of *β*-Ag_2_Te can convert the nontrivial topologically insulating phase into a trivial semiconducting phase. The *β*-Ag_2_Te-based TPT-FET devices with a simple top-gate device configuration were successfully fabricated via a standard device fabrication process, showing excellent device-to-device reproducibility. Although challenges remain in areas such as operating temperature improvement and process integration, the universality of its physical mechanism points the way for exploring richer material systems (for example, magnetic topological materials, few-layer topological semimetal, two-dimensional topological insulators) in future research. We believe that through the synergistic optimization of materials science, device physics, and micro-nano fabrication technology, TPT-FETs are poised to become a promising candidate technology for future low-power, high-performance electronics.

## METHODS

### CVD growth and characterization of *β*-Ag_2_Te nanoplates

High quality 2D *β*-Ag_2_Te nanoflakes were synthesized according to previously described methods. The bulk of Te (Alfa Aesar, 0.5 g) and the mixture of Bi_2_O_3_ (Macklin, 1 g) and AgCl (Damas, 0.1 g) were placed in the upstream and downstream heating zones, respectively. The mica substrates were located close to the downstream heating center (1–3 cm away). To obtain the thinner *β*-Ag_2_Te nanoflakes, the evaporated temperatures of Te and mixture of Bi_2_O_3_ + AgCl were kept at 420 and 620°C, and 200 sccm Ar was employed as the carrier gas. The pressure was kept constant at 400 torr during the whole CVD growth process.

To characterize the as-grown *β*-Ag_2_Te nanoflakes, optical microscopy (OM, WY-910), atomic force microscopy (AFM, Bruker Dimension Icon), and transmission electron microscopy (TEM, FEI F20) were used.

### Device fabrication and electrical transport measurements

The top-gate six-terminal Hall-bar devices were fabricated by the following process. First, the standard location markers were pre-patterned on mica substrate by UV photolithography. Then, electron-beam lithography (EBL) was used to write six electrode legs of the Hall bar device on CVD-grown *β*-Ag_2_Te, followed by thermal evaporation of the contact metals Pd/Au (5/30 nm). The Al_2_O_3_ (12 nm) dielectric was deposited by ALD at 100°C. The top-gate electrodes were fabricated by another step EBL and thermal evaporation of Pd/Au (5/30 nm).

The quantum oscillations and transfer curves were collected in the Physical Properties Measurement Systems (PPMS-9T, Quantum Design) coupled with a home-made electrical measurement system, composed of two Keithley 2182A nanovoltmeters, a Keithley 6221 AC and DC current source, and two Keithley 2400 meters. The magnetic field is applied perpendicularly to the device plane.

### Theoretical method

First-principles calculations are performed with the framework of DFT, as implemented in the Vienna Ab Initio Simulation Package. The exchange-correlation functional adopts the generalized gradient approximation based on the Perdew–Burke–Ernzerhof formulation. The kinetic energy cutoff is set as 450 eV. For bulk *β*-Ag_2_Te optimization, an 8 × 12 × 8 Monkhorst–Pack k-point mesh is used for Brillouin zone integration. The criterion for full structural relaxation is the Hellmann–Feynman forces reduce below 0.001 eV/Å and the total energy difference decrease smaller than 10^−6^ eV in iteration. For the surface calculations, we construct a slab model using a 1 × 1 × 12 supercell of *β*-Ag_2_Te. To minimize periodic cell interactions, a 30 Å vacuum layer is set along *z*-axis. We passivate all dangling bonds at the slab surfaces using hydrogen atoms to stabilize geometry of the slab and prevent surface reconstruction. External electric fields are applied along the [001] direction from 0.02 to 0.1 eV/Å. The electronic calibration maintains the above convergence criteria under electric fields. Band structure calculations are performed with SOC. The surface states are calculated by a tight-binding model constructed using WANNIER90 package.

## Supplementary Material

nwag229_Supplemental_File
