# Supplementary material for: Transport signatures of gate-tunable topological phase transition in ultrathin β-Ag2Te
Source: Natl Sci Rev. 2026 Apr 17;13(12):nwag229. doi: 10.1093/nsr/nwag229 (PMC13274656; doi:10.1093/nsr/nwag229)
Supplement: nwag229_Supplemental_File [file nwag229_supplemental_file.docx]

**Supplementary Materials:**

**Transport signatures of gate-tunable topological phase transition in ultrathin *β*-Ag_2_Te**

Wei Ai ^1, †^, Xiao-Feng Luo^2, 3, †^, Zhaochao Liu^1^, Ying Deng^4^, Zunxian Lv^1^, Yuyu He^1^, Lingyue Li^1^, Xuewen Fu^4^, Feng Luo^1,^ *, Jingyue Wang^5,^ *, Jin-Zhu Zhao^2, 3, 6,^ *, Jinxiong Wu^1,^ *

*^1^Tianjin Key Lab for Rare Earth Materials and Applications, Center for Rare Earth and Inorganic Functional Materials, School of Materials Science and Engineering, Nankai University, Tianjin 300350, China;*

*^2^Guangdong Basic Research Center of Excellence for Structure and Fundamental Interactions of Matter, Guangdong Provincial Key Laboratory of Quantum Engineering and Quantum Materials, School of Physics, South China Normal University, Guangzhou 510006, China;*

*^3^Guangdong-Hong Kong Joint Laboratory of Quantum Matter, Frontier Research Institute for Physics, South China Normal University, Guangzhou 510006, China;*

*^4^Ultrafast Electron Microscopy Laboratory, Key Laboratory of Weak-Light Nonlinear Photonics, School of Physics, Nankai University, Tianjin 300071, China;*

*^5^Shandong Key Laboratory of Intelligent Energy Materials, School of Materials Science and Engineering, China University of Petroleum (East China), Qingdao 266580, China;*

*^6^Center for Computational Science and Engineering, Southern University of Science and Technology, Shenzhen 518055, China.*

***Corresponding authors**. E-mail: jxwu@nankai.edu.cn; zhaojz@m.scnu.edu.cn; jingyue_wang@upc.edu.cn; feng.luo@nankai.edu.cn.

^†^Equally contributed to this work.

1. Theoretical method

To simulate the electric field-induced Stark effect, we applied electric fields along the [001] direction incrementally from 0.00 eV/Å to 0.10 eV/Å, divided into 10 steps with an increment of 0.01 eV/Å. This incremental approach was chosen to ensure computational stability. In topological materials, directly applying a large electric field may lead to numerical instabilities, such as abrupt changes in charge density distribution or convergence failures. By using small increments, we allow the system to gradually adapt to the electric field, mitigating computational artifacts that could arise from transitioning directly from zero to high fields, thereby enhancing the reliability and accuracy of the results. This strategy has been widely adopted in previous studies of field-tuned topological phase transitions, such as those simulating the Stark effect in two-dimensional materials, to prevent non-physical oscillations or convergence issues in self-consistent field calculations.

The calculations for each electric field value (*E*-field) were performed using the following detailed procedure:

1. Structural Relaxation without *E*-Field.

Initially, the slab model under zero electric field was relaxed until the Hellmann-Feynman forces on each atom were below 0.001 eV/Å and the energy convergence criterion was below 10^-6^ eV. This stringent convergence standard ensures that the initial structure is at its energetic minimum, eliminating residual stresses that could affect the accuracy of subsequent electric field calculations.

2. Self-Consistent field (SCF) Calculation at 0.01 eV/Å *E*-Field

Using the relaxed structure obtained above, the SCF calculations were performed for an electric field of 0.01 eV/Å with spin-orbital-coupling (SOC) effect included. This step facilitates a smooth transition from zero to a small electric field, avoiding issues associated with sudden charge redistribution. The SCF calculations iteratively solve the Kohn-Sham equations until the electron density and total energy converge, using the same 10^-6^ eV energy criterion.

3. Calculations for Incremental Higher *E*-Fields

For subsequent electric field values (starting from 0.02 eV/Å), the wavefunction and charge density files from the previous step were used as initial inputs for the SCF calculations in the present of SOC effect. This “inheritance” approach leverages the optimized results from the prior step as a robust initial guess, significantly accelerating convergence and maintaining computational continuity. Directly recalculating from the zero-field state at higher fields may lead to discontinuities in electron density or unphysical polarization, introducing non-physical results. This method has been wildly confirmed and reported in series literatures, for instance, in studies of the giant Stark effect in black phosphorus [1] or transition metal dichalcogenides (TMDs) [2], where it ensures numerical stability and reproducibility of band gap modulation.

4. Band Structure Calculations

After completing the SCF calculations for each electric field, the band structures were obtained via non-self-consistent calculations, using the updated wavefunction and charge density files as the input. In this step, the effect of SOC is considered as well.

Similar incremental strategies have been employed in studies of field-tuned topological phases to avoid numerical instabilities.

1. Sample characterization


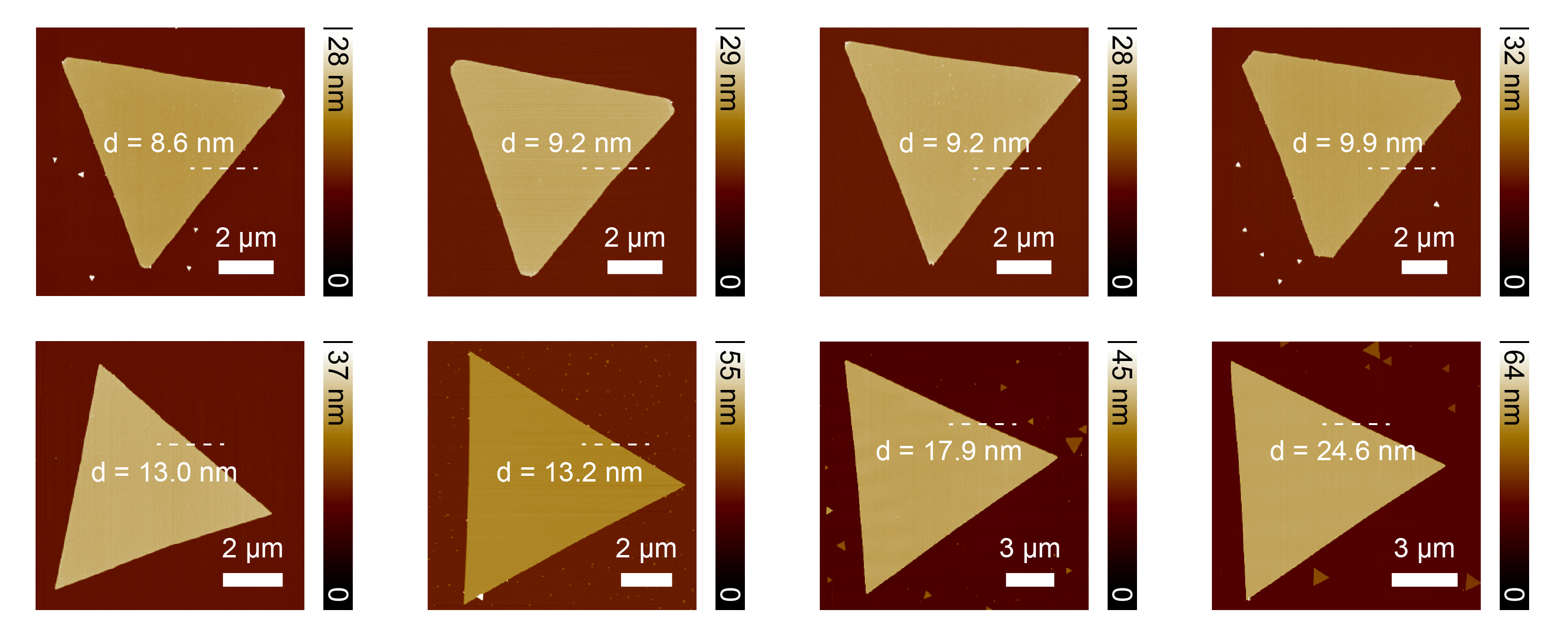


**Figure S1.** AFM images of ultrathin *β*-Ag_2_Te nanosheets grown at low temperature (893 K), showing a typical thickness in the range of 8-25 nm.


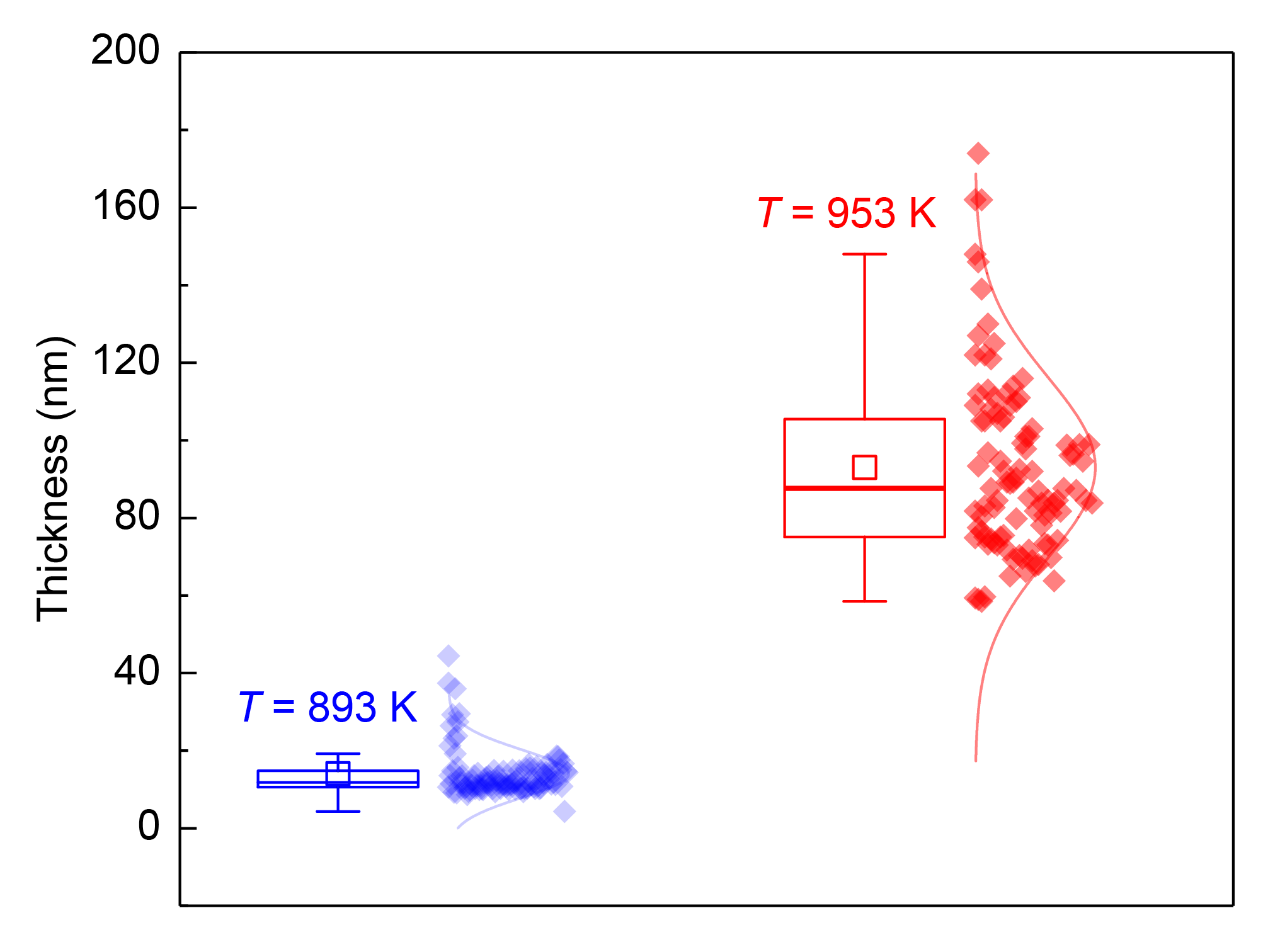


**Figure S2.** The thickness statistic for *β*-Ag_2_Te nanosheets grown at different temperatures (The blue refers to 893 K, and the red corresponds to 953 K). Typically, the *β*-Ag_2_Te nanosheets grown at 893 K are thinner than the one grown at 953 K.


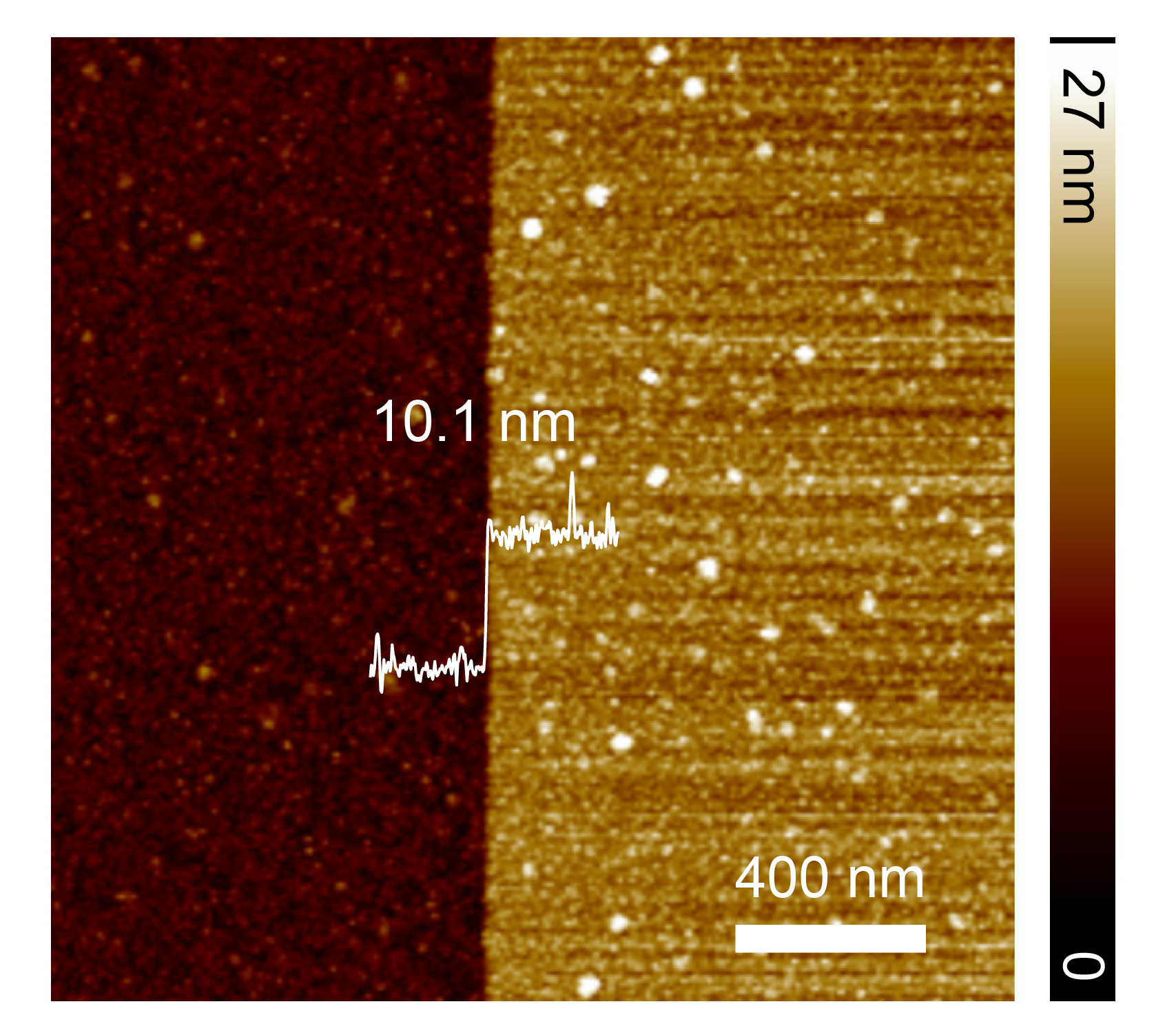


**Figure S3.** The AFM image and height profile of *β*-Ag_2_Te device in Figure 2 (main text), showing a thickness of 10.1 nm.

1. **Transport measurements on *β*-Ag_2_Te top-gate device**

Lifshitz-Kosevich formula is the best way to describe the quantum oscillations at low temperatures.

$\begin{aligned} \frac{\Delta R(B)}{R(0)}\propto\sum_{p} {A_{p}R}_{S}{\left( p \right)R}_{T}\left( p \right)R_{D}\left( p \right)\cos\left( 2\pi\left( \frac{B_{f}}{B}+\gamma\right) \right)\#\left( A1 \right) \end{aligned}$

As shown in Equation A1, the total quantum oscillations of a Fermi surface are composed of the sum of quantum oscillations with different harmonics (*p* = 1, 2, 3, 4…). Figure S6 is the FFT analysis of the quantum oscillations in Figure 2(e). Interestingly, the fundamental (1^st^) frequency of quantum oscillations lays in f ≈ 22 T, and the positions of another two peaks are about 47 T and 84 T, which are very close to the 2^nd^ and 4^th^ harmonic. We would like to emphasize that the high harmonic generations of quantum oscillations are quite common in high-mobility systems [3-5].


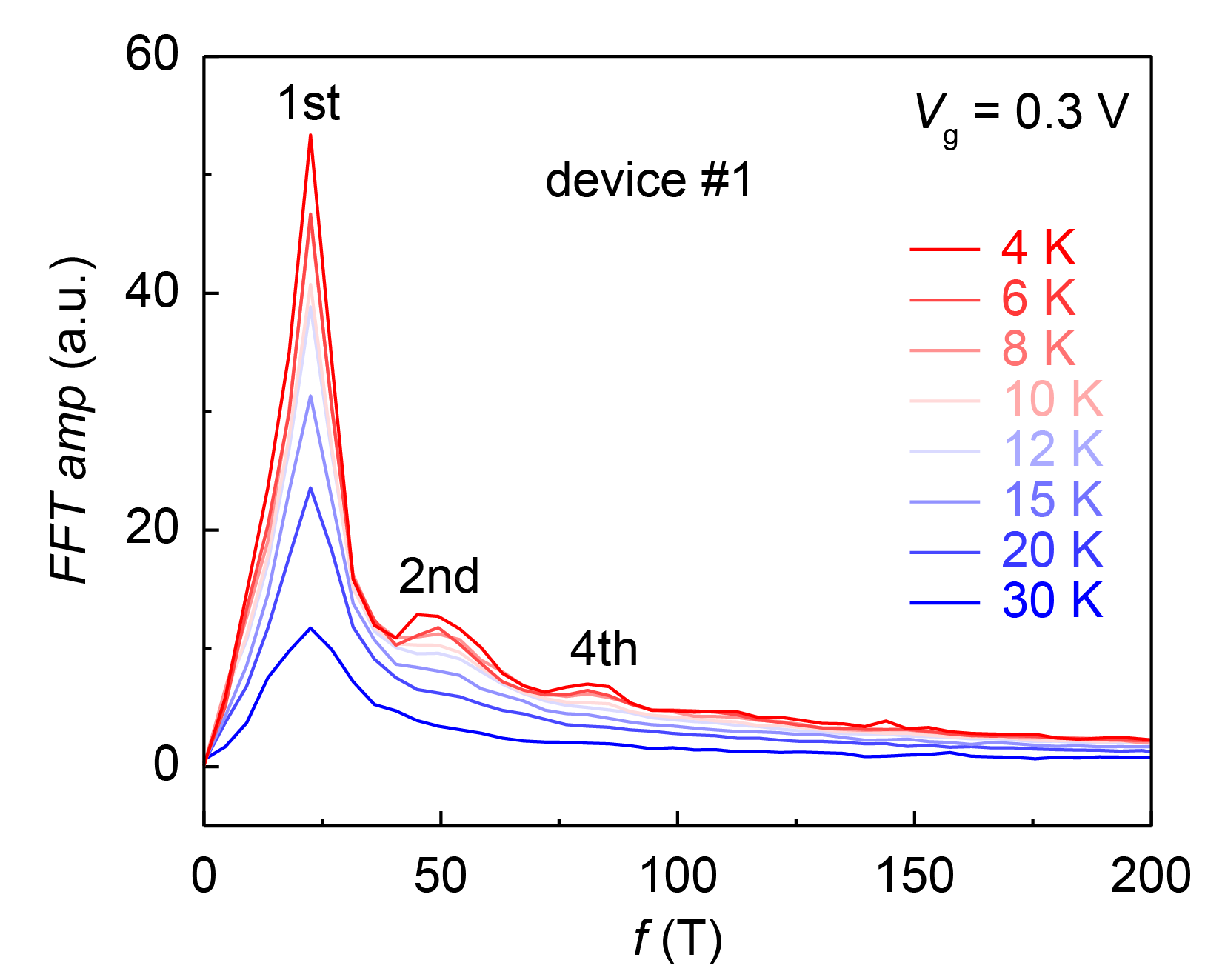


**Figure S4.** Temperature-dependent FFT analysis of SdH oscillations in Figure 2e (device #1), showing fundamental frequency (1^st^), 2^nd^ and 4^th^ harmonic at low temperature.


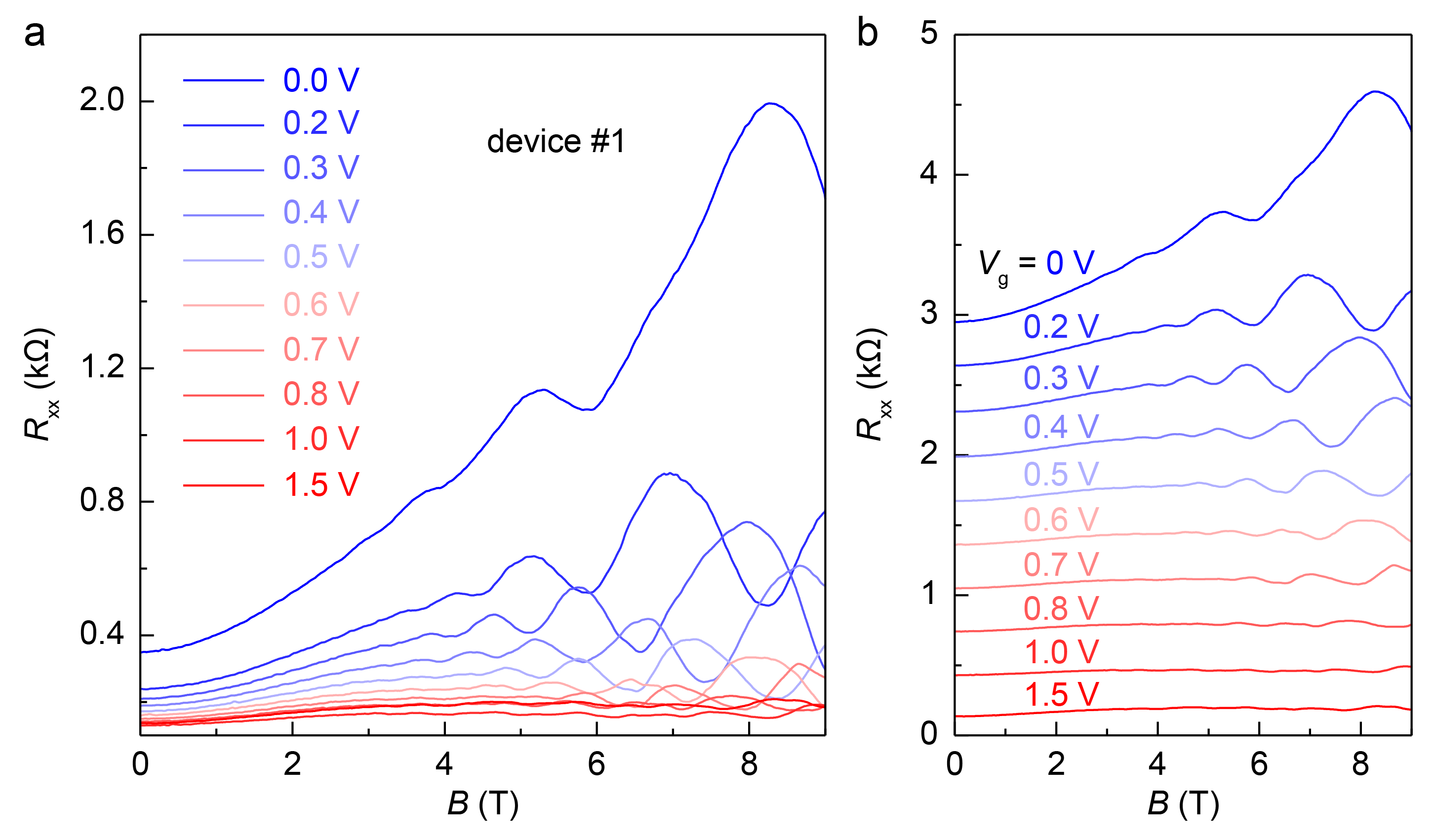


**Figure S5.** Gate-dependent magnetoresistance corresponding to Figure 3a (device #1). (a) The raw magnetoresistance. (b) The curves are vertically shifted by 300 Ω. The raw MR data clearly exhibits SdH oscillations. Both the frequency and amplitude of these oscillations evolve systematically with the applied gate voltage from 0 to 1.5 V, providing direct evidence of the changing Fermi surface. Here, we should emphasize that applying negative voltages will drive the system into off state with a large resistance rapidly, which will make it difficult to form excellent Ohmic contact during Hall measurements, especially at high magnetic field.


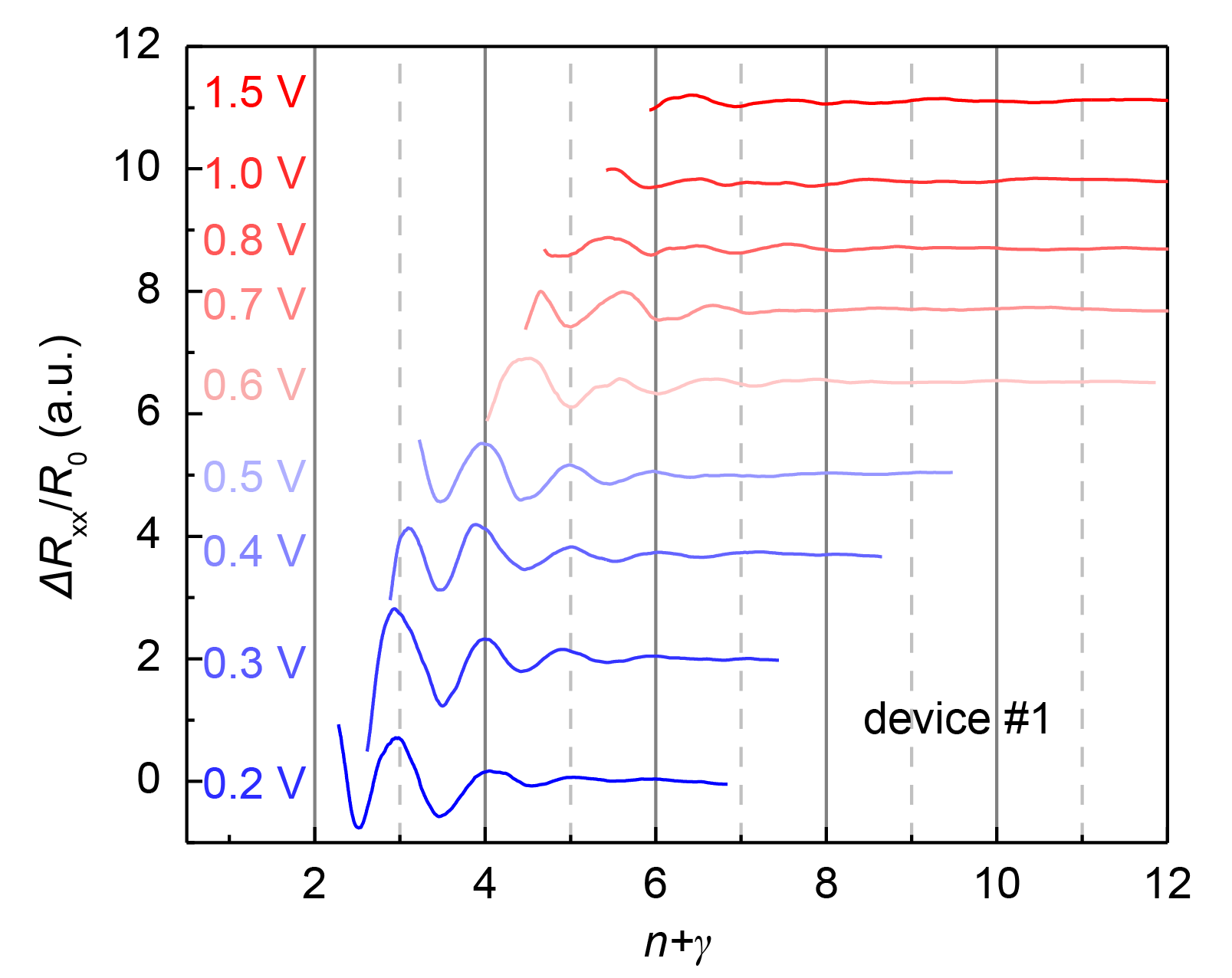


**Figure S6.** Oscillations as a function of *n+γ* under varying external gate-voltage in device #1, showing a significant peak-to-valley transition when the gate voltage changes from 0.5 V to 0.6 V.


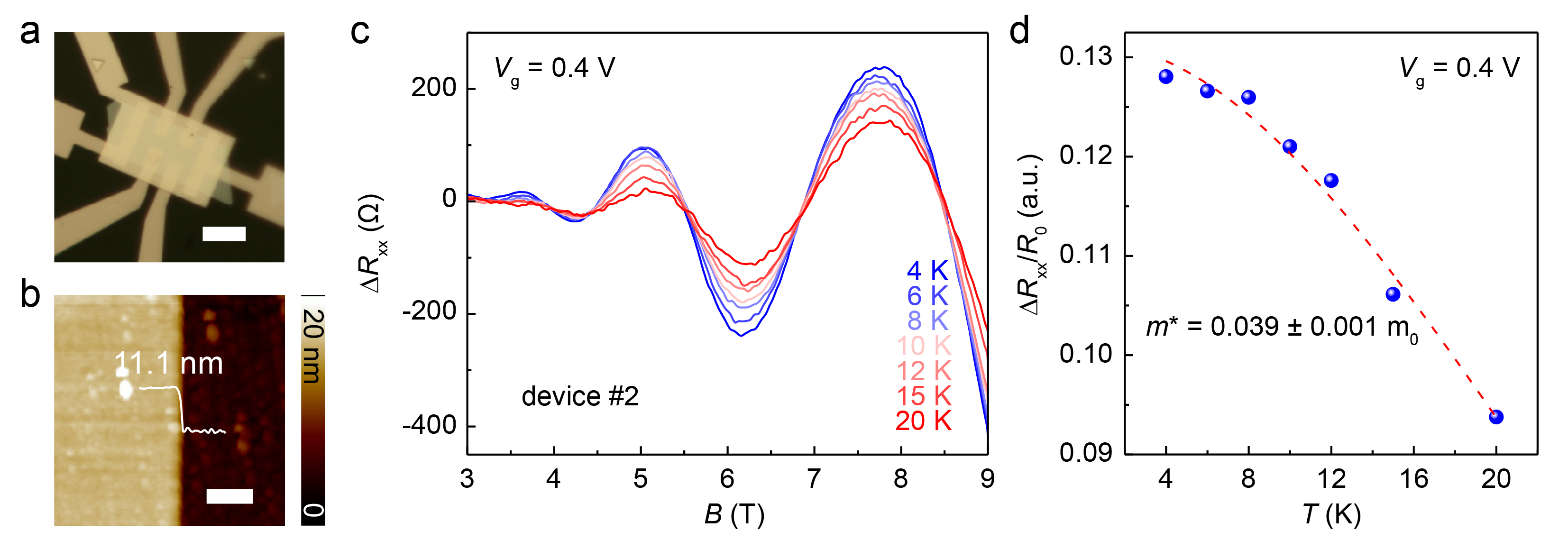


**Figure S7.** Shubnikov-de Haas quantum oscillations of another as-synthesized ultrathin *β*-Ag_2_Te crystal with a thickness of 11.1 nm (device #2). (a) OM image of the as-fabricated top-gate Hall-bar device. Bar: 10 μm. (b) The corresponding AFM image and height profile, showing a thickness of 11.1 nm. Bar: 400 nm. (c) Temperature-dependent SdH oscillations amplitude (subtracting a smooth background) from 4 K to 20 K. (d) Temperature-dependent Δ*R*_xx_/*R*_0_ values of the SdH oscillations, showing a very small average value of 0.039 ± 0.001 m_0_.


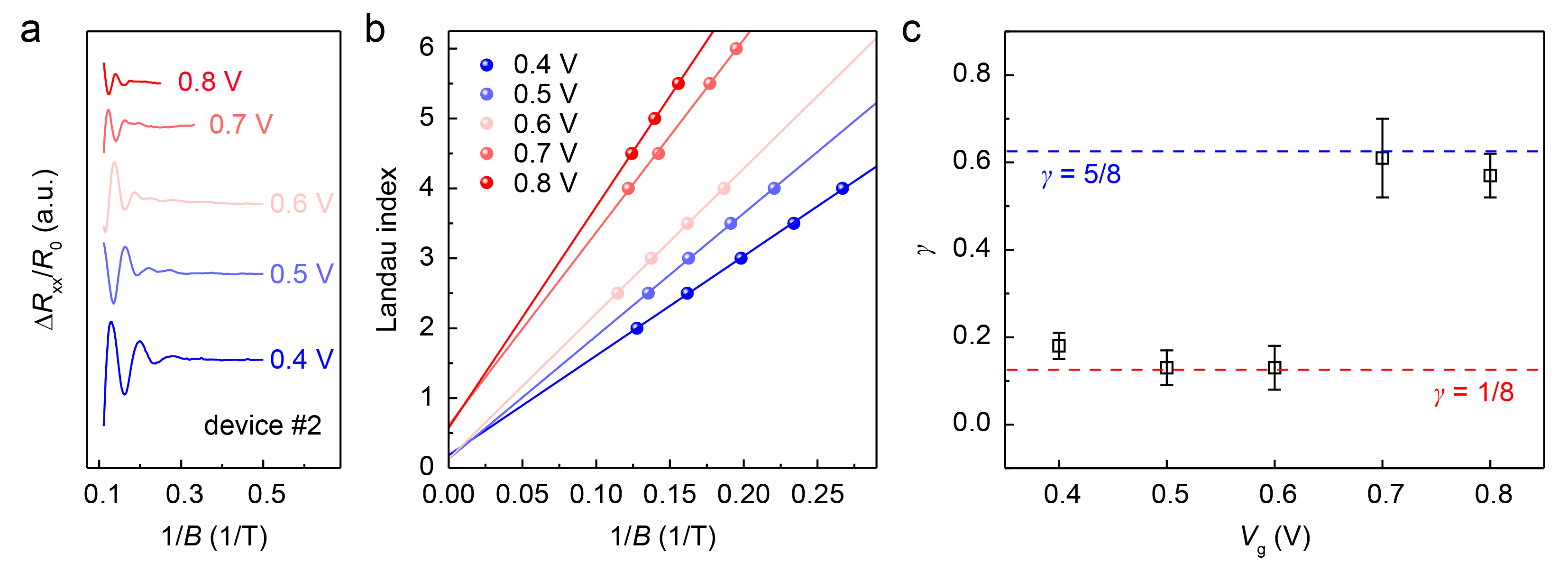


Figure S8. Gate-tunable topological phase transition in ultrathin *β*-Ag_2_Te crystal with a thickness of 11.1 nm (device #2). (a) A stacking view of SdH oscillations amplitude as a function of 1/*B* at various gate voltage. (b) Landau fan diagram at various top-gate voltage. Where an integer Landau index is assigned to the peak of SdH oscillation and a half-integer is assigned to the valley. (c) The intercept *γ* of Landau fan diagram as a function of top-gate voltage. A sudden change from 1/8 to 5/8 occurs at a critical voltage, indicating a clear gate-induced topological phase transition.


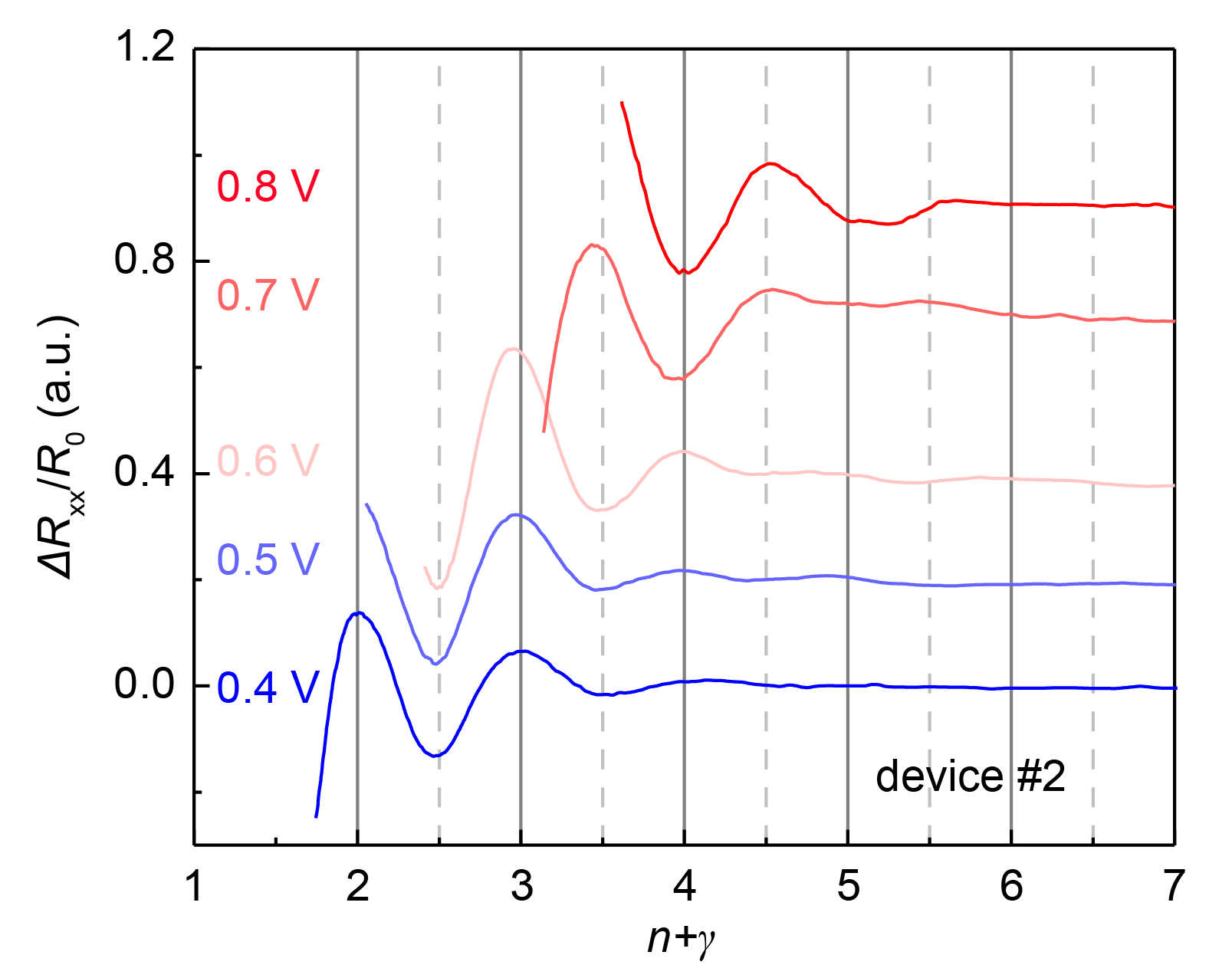


**Figure S9.** Oscillations as a function of *n+γ* under varying external gate voltages in device #2, showing a significant peak-to-valley transition when the gate voltage changes from 0.6 V to 0.7 V.

7


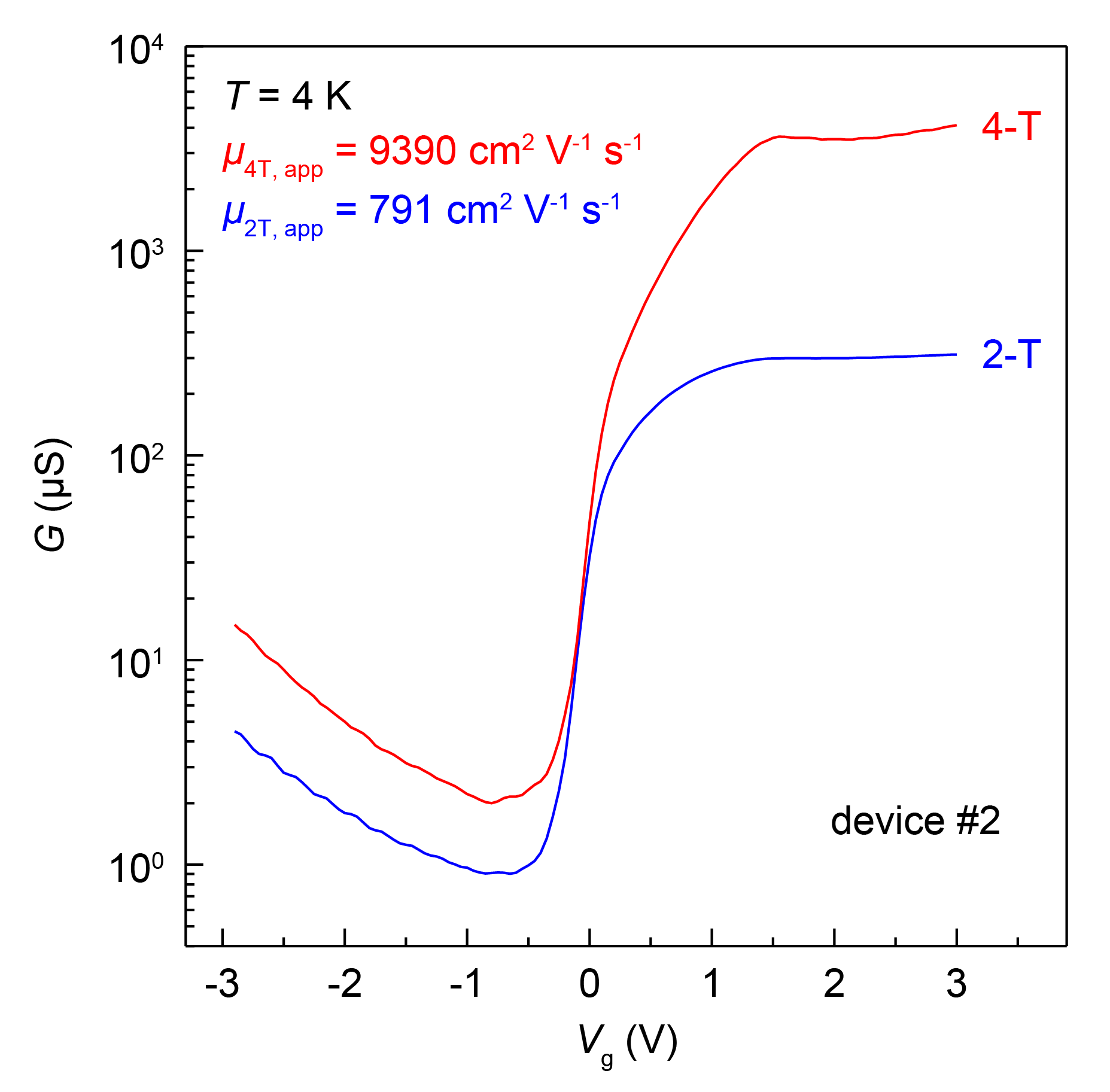


**Figure S10.** The comparison of 2-probe transfer curve and 4-probe transfer curve of a *β*-Ag_2_Te-based TPT transistor with a thickness of 11.1 nm (device #2).

To investigate the dimensional character of the quantum oscillations, we further fabricated new top-gated six-terminal devices and performed detailed transport measurements. As shown in Figure S11, a topological phase transition occurs at a gate voltage of approximately 2.4 V in a sample with a thickness of about 13.9 nm.

For a two-dimensional Fermi surface, the motion of electrons is confined within the plane. When a magnetic field was applied at an angle *θ* to the normal direction of the 2D plane, the component that truly affects the 2D electron gas is the perpendicular magnetic field component, *B*_⊥_ = *B* × cos*θ*. Only this component, which is perpendicular to the 2D plane, can quantize the in-plane motion of electrons into Landau levels. Therefore, when *B* × cos*θ* is used as the horizontal coordinate, all the oscillation peaks and valleys will perfectly coincide onto a single curve.

To investigate the dimensional character of the quantum oscillations, we further performed detailed angle-dependent SdH measurements. The angular dependent quantum oscillations before and after the phase transition are presented in Figure S12. With the horizontal coordinate representing *B* × cos*θ* (i.e., the equivalent out-of-plane magnetic field), the positions of the peaks and valleys in the SdH oscillations remain unchanged during the rotation process, confirming the two-dimensional characteristics of *β*-Ag_2_Te. Combined with the observed non-trivial Berry phase in the low gate voltage regime, it indicates that the quantum oscillations originate from the surface states.

However, after the phase transition (under higher gate voltage), the angle-dependent oscillations still exhibit a 2D characteristic. The 2D nature of quantum oscillations in the topologically nontrivial regime originates from the thin thickness of our sample, similar to the case found in typical semiconductors, such as Bi_2_O_2_Se and black phosphorus [6-8].


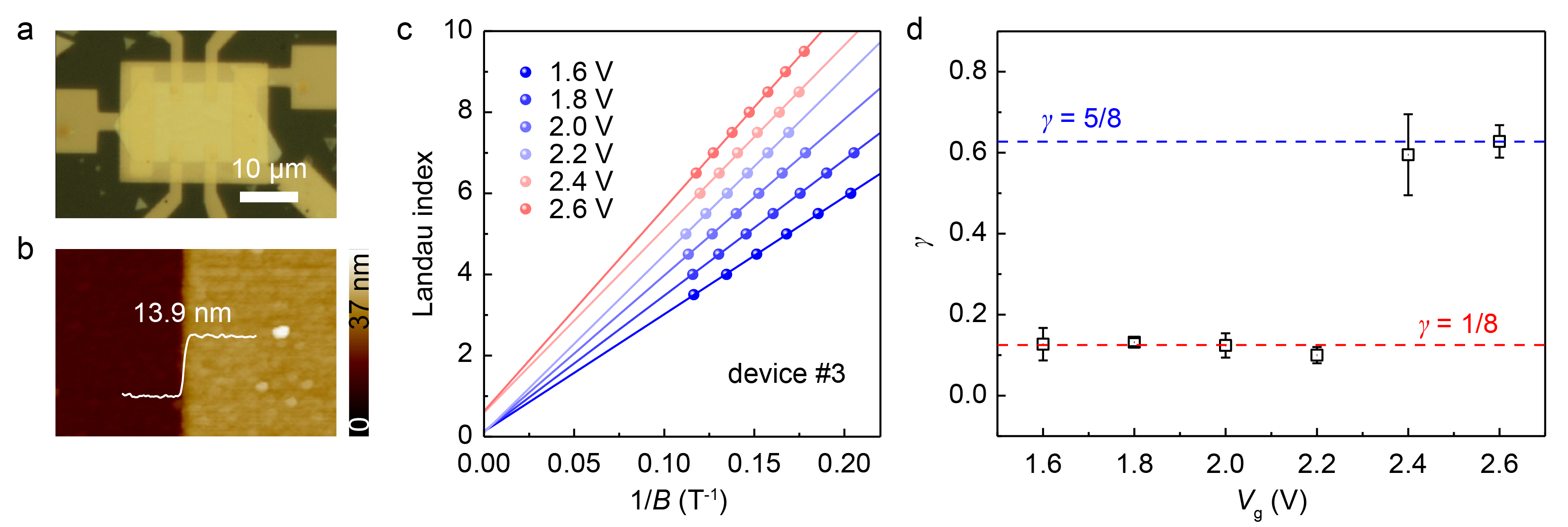


**Figure S11.** Gate-tunable topological phase transition in 13.9-nm-thick *β*-Ag_2_Te nanoflake (device #3). (a) OM image of the as-fabricated top-gate Hall bar device. (b) Corresponding AFM image and height profile, showing a thickness of 13.9 nm. (c) Landau fan diagram at various top-gate voltage. Where an integer Landau index is assigned to the peak of SdH oscillation and a half-integer is assigned to the valley. (d) The intercept γ of Landau fan diagram as a function of top-gate voltage. A sudden change from 1/8 to 5/8 occurs at a critical voltage, indicating a clear topological phase transition.


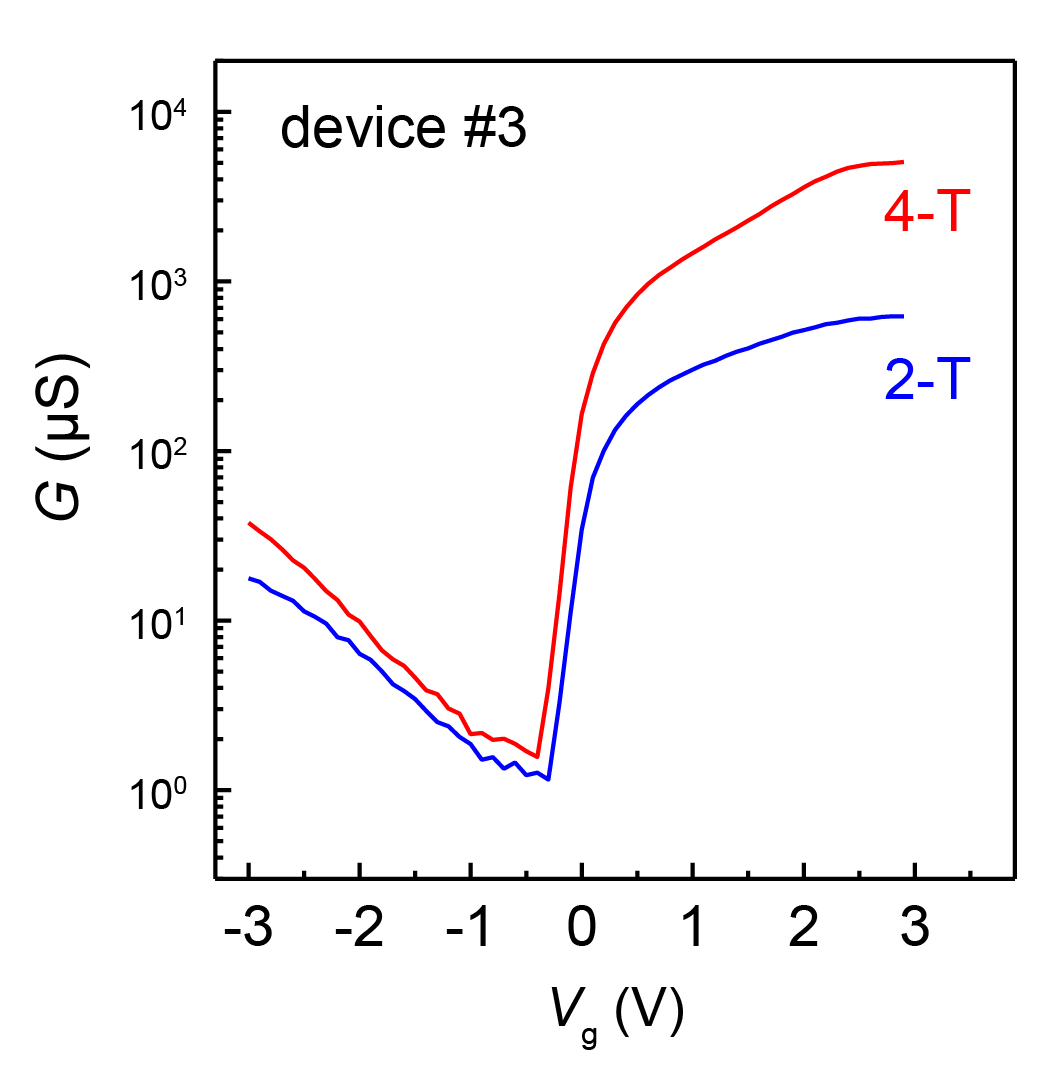


**Figure S12.** The comparison of 2-probe transfer curve and 4-probe transfer curve of a *β*-Ag_2_Te-based TPT transistor with a thickness of 13.9 nm (device #3).


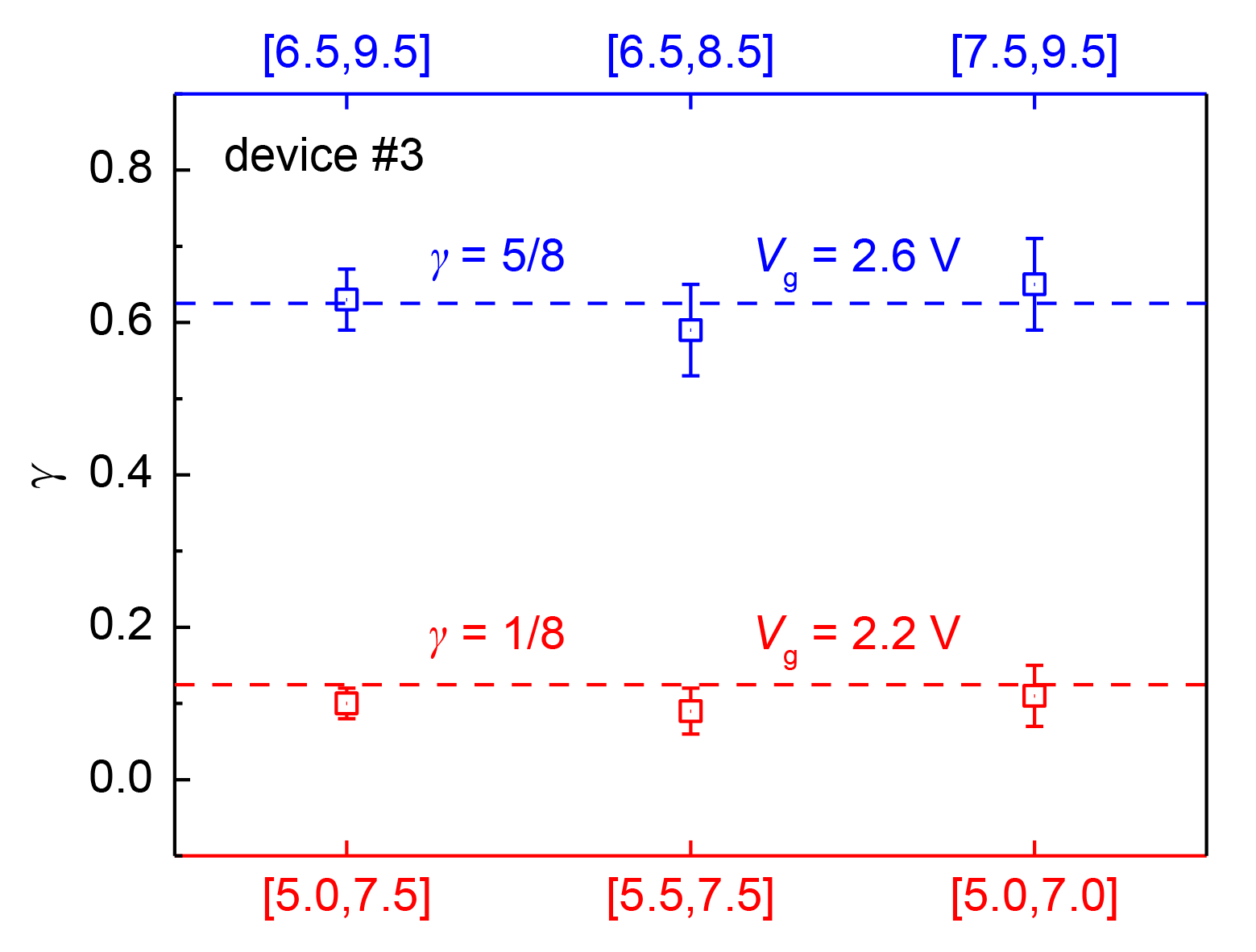


**Figure S13.** The variation of the extracted intercept with the fitting range before and after the phase transition in device #3. The x-coordinate [x, y] means the range of Landau level index used for linear fitting.


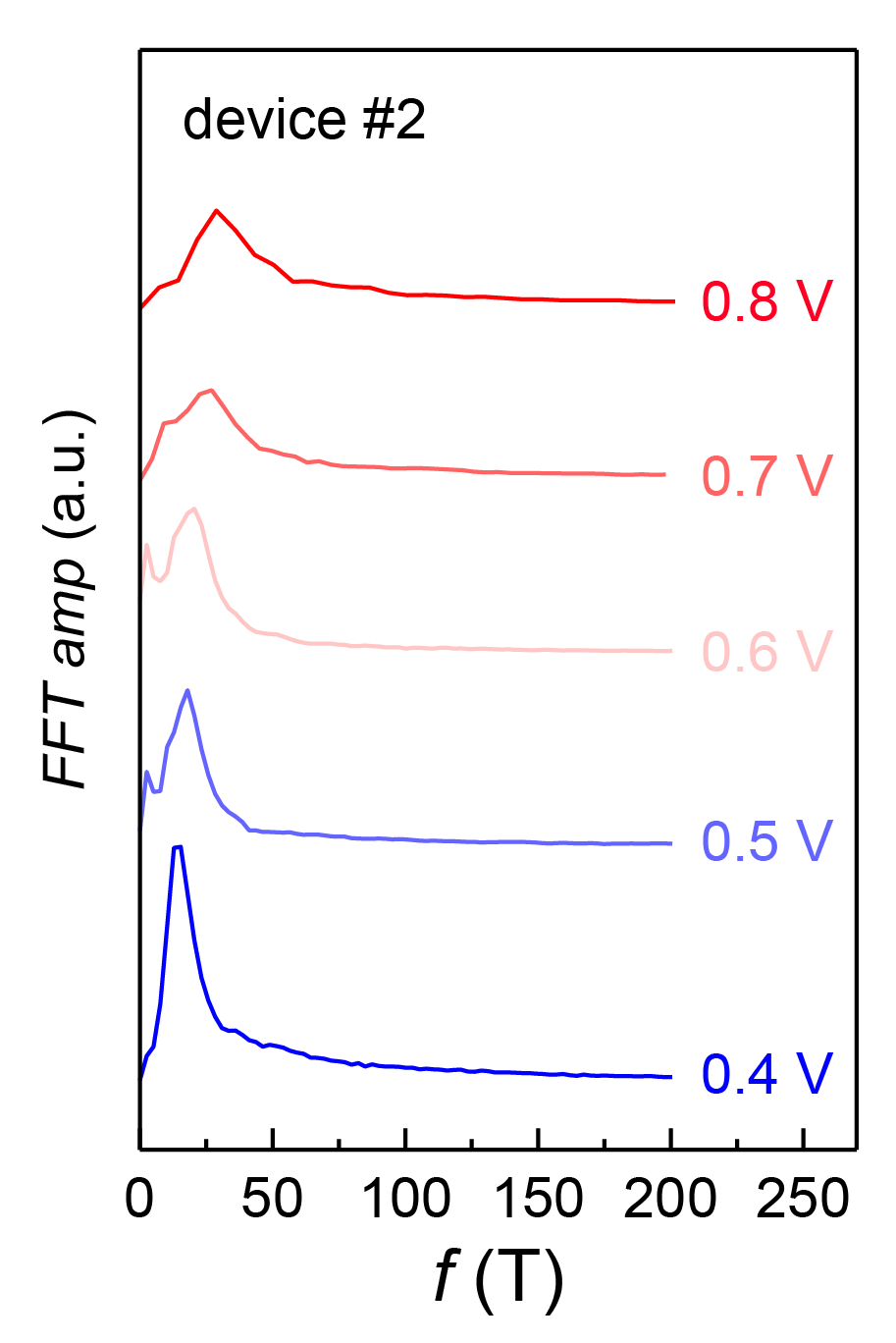


**Figure S14.** FFT spectrum of the SdH oscillations in device #2, exhibiting a single-band nature near critical gate voltage.


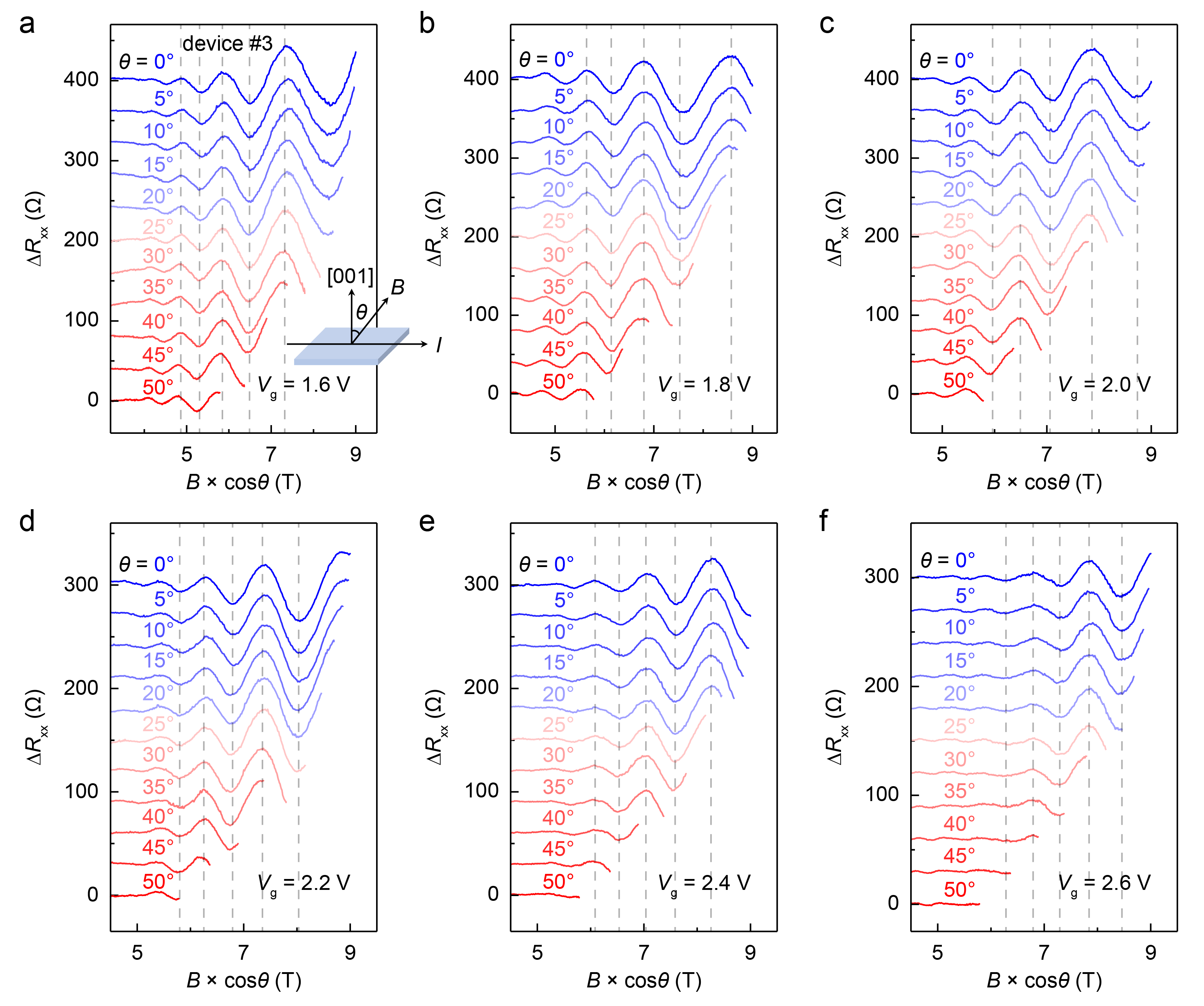


**Figure S15.**  Angle-dependent SdH oscillations as a function of effective magnetic fields (*B*_⊥_, *B*_⊥_ = *B* × cos*θ*, where *B* is the applied magnetic field) under various top-gate voltage (*V*_g_) in 13.9-nm-thick *β*-Ag_2_Te nanoflakes (device #3). (a) *V*_g_ = 1.6 V. (b) *V*_g_ = 1.8 V. (c) *V*_g_ = 2.0 V. (d) *V*_g_ = 2.2 V. (e) *V*_g_ = 2.4 V. (f) *V*_g_ = 2.6 V. The inset show *θ* as the angle between the magnetic field and the normal direction of the sample plane. The curves in (a), (b) and (c) are vertically shifted by 40 Ω, the curves in (d), (e) and (f) are vertically shifted by 30 Ω.


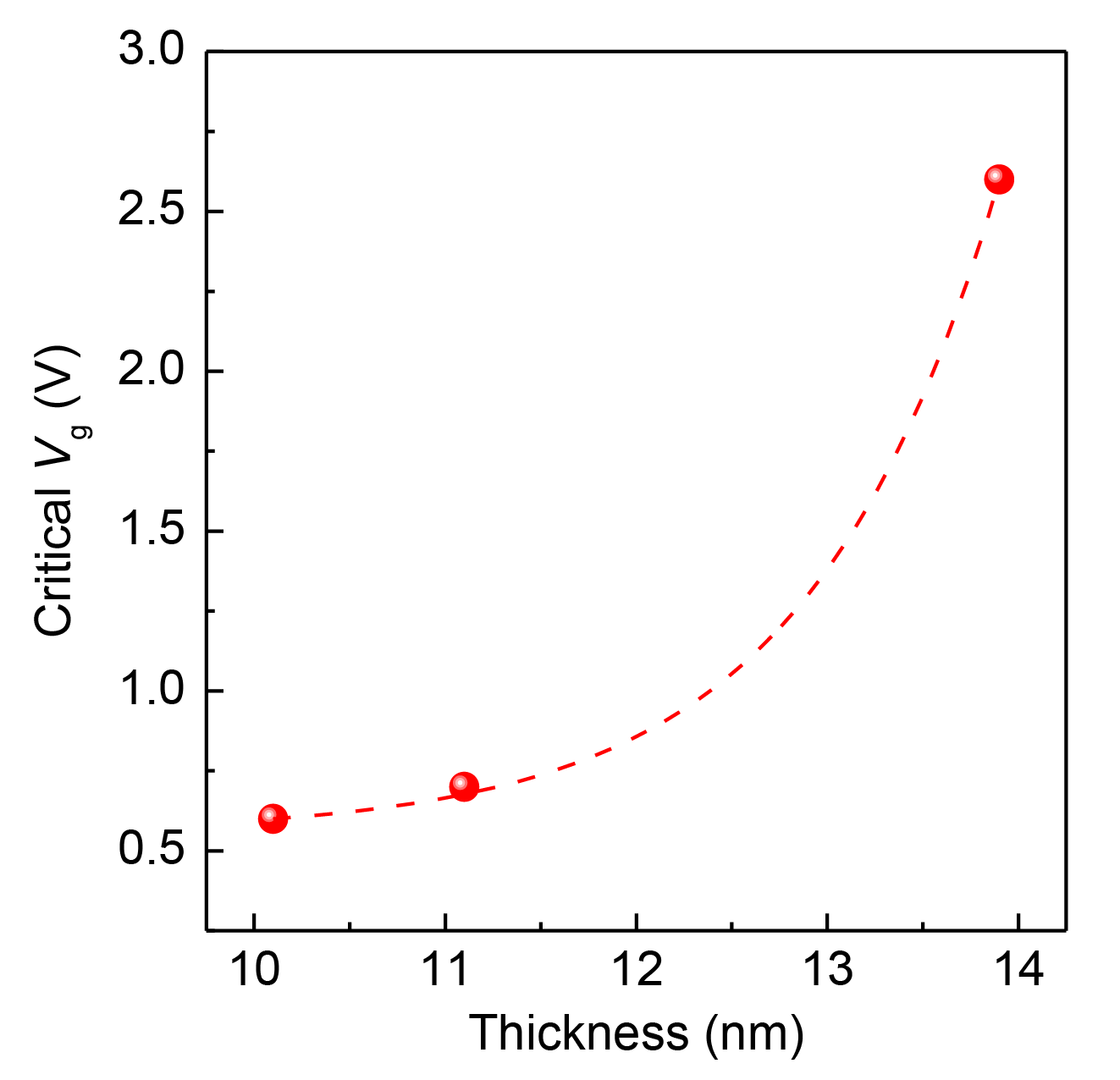


**Figure S16.** The critical gate voltage as a function of sample thickness, showing a clear increasing trend.


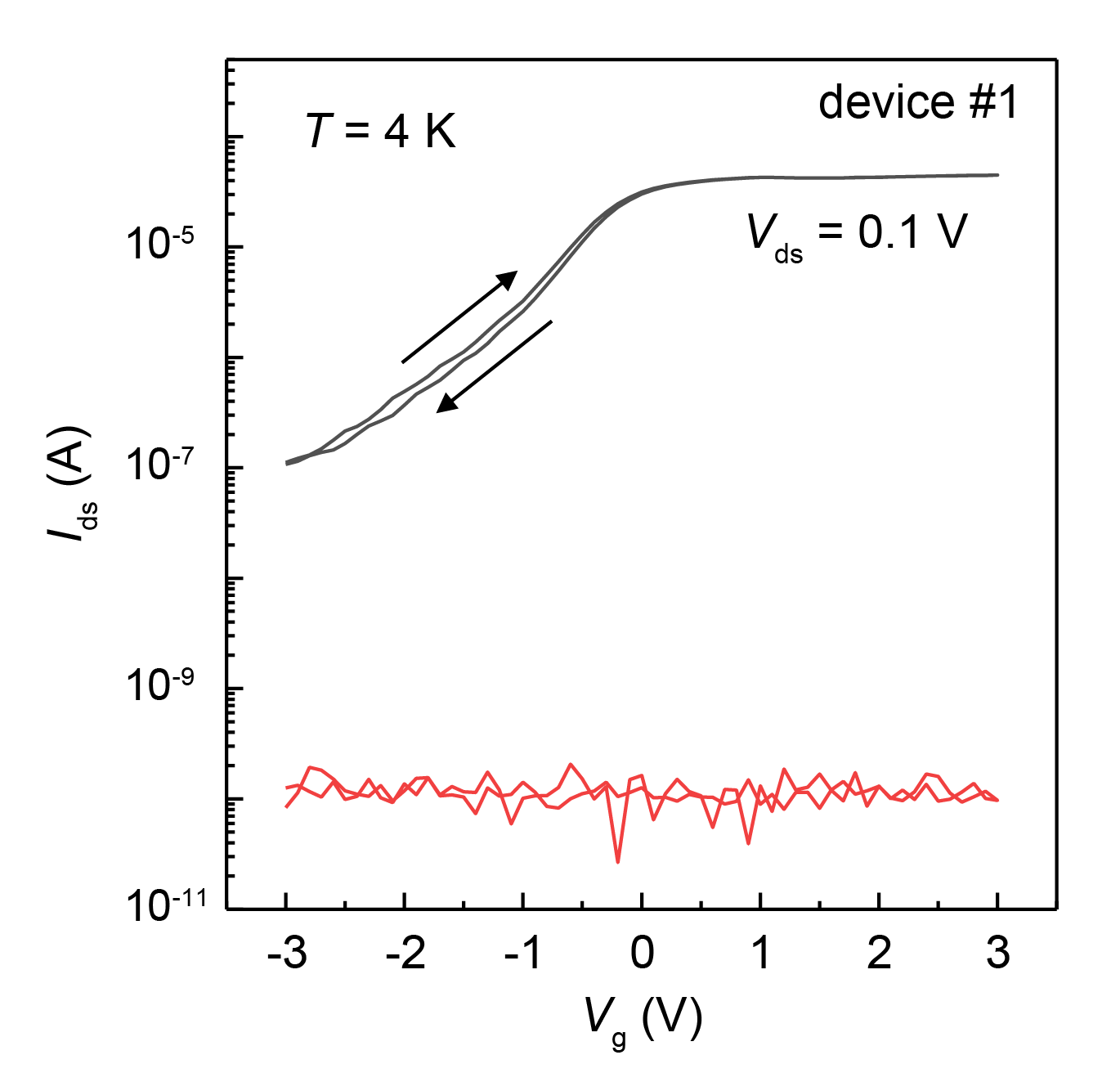


**Figure S17.** Two-probe transfer curve of device #1 at 4 K, showing a slight clockwise hysteresis.

**REFERENCES**

1. Liu, Y., Qiu, Z., Carvalho, A., Bao, Y., Xu, H., Tan, S. J. R., Liu, W., Castro Neto, A. H., Loh, K. P. & Lu, J. Gate-tunable giant Stark effect in few-layer black phosphorus. *Nano Lett.* **17**, 1970-1977 (2017).

2. Murthy, A. A., Stanev, T. K., dos Reis, R., Hao, S., Wolverton, C., Stern, N. P. & Dravid, V. P. Direct visualization of electric-field-induced structural dynamics in monolayer transition metal dichalcogenides. *ACS Nano* **14**, 1569-1576 (2020).

3. Juraszek, J., Bochenek, L., Rudenko, A., Hosen, M. M., Daszkiewicz, M., Wang, Z., Wosnitza, J., Henkie, Z., Samsel-Czekała, M., Neupane, M. & Cichorek, T. Nonsaturating extreme magnetoresistance and large electronic magnetostriction in LuAs. *Phys. Rev. Res.* **1**, 032016 (2019).

4. Sheng, F., Hua, C., Cheng, M., Hu, J., Sun, X., Tao, Q., Lu, H., Lu, Y., Zhong, M., Watanabe, K., Taniguchi, T., Xia, Q., Xu, Z.-A. & Zheng, Y. Rashba valleys and quantum Hall states in few-layer black arsenic. *Nature* **593**, 56-60 (2021).

5. Feng, Y., Wang, Y., T. F., R., P. B., L. & Chen, H. Quantum interference in superposed lattices. *Proc. Natl. Acad. Sci.* **121(7)**, e2315787121 (2024).

6. Li, L., Ye, G. J., Tran, V., Fei, R., Chen, G., Wang, H., Wang, J., Watanabe, K., Taniguchi, T., Yang, L., Chen, X. H. & Zhang, Y. Quantum oscillations in a two-dimensional electron gas in black phosphorus thin films. *Nat. Nanotechnol.* **10**, 608-613 (2015).

7. Wang, J., Huang, J., Kaplan, D., Zhou, X., Tan, C., Zhang, J., Jin, G., Cong, X., Zhu, Y., Gao, X., Liang, Y., Zuo, H., Zhu, Z., Zhu, R., Stern, A., Liu, H., Gao, P., Yan, B., Yuan, H. & Peng, H. Even-integer quantum Hall effect in an oxide caused by a hidden Rashba effect. *Nat. Nanotechnol.* **19**, 1452-1459 (2024).

8. Tsui, D. C., Stormer, H. L. & Gossard, A. C. Two-dimensional magnetotransport in the extreme quantum limit. *Phys. Rev. Lett.* **48**, 1559-1562 (1982).
